# Adult dental epithelial stem cell-derived organoids deposit hydroxylapatite biomineral

**DOI:** 10.1038/s41368-023-00257-w

**Published:** 2023-12-07

**Authors:** Hyun-Yi Kim, Victoria Cooley, Eun-Jung Kim, Shujin Li, Jong-Min Lee, Dina Sheyfer, Wenjun Liu, Ophir D. Klein, Derk Joester, Han-Sung Jung

**Affiliations:** 1 NGeneS Inc., Ansan-si, Gyeonggi-do Korea; 2 Department of Materials Science and Engineering, Northwestern University, Evanston, IL USA; 3 X-ray Science Division, Advanced Photon Source, Argonne National Laboratory, Lemont, IL USA; 4 Division in Anatomy and Developmental Biology, Department of Oral Biology, Oral Science Research Center, BK21 FOUR Project, Yonsei University College of Dentistry, Seoul, Korea; 5 Department of Orofacial Sciences and Program in Craniofacial Biology, University of California, San Francisco, CA USA; 6 Department of Pediatrics, Cedars-Sinai Guerin Children’s, Los Angeles, CA USA

**Keywords:** Adult stem cells, Stem-cell research

## Abstract

Ameloblasts are specialized cells derived from the dental epithelium that produce enamel, a hierarchically structured tissue comprised of highly elongated hydroxylapatite (OHAp) crystallites. The unique function of the epithelial cells synthesizing crystallites and assembling them in a mechanically robust structure is not fully elucidated yet, partly due to limitations with in vitro experimental models. Herein, we demonstrate the ability to generate mineralizing dental epithelial organoids (DEOs) from adult dental epithelial stem cells (aDESCs) isolated from mouse incisor tissues. DEOs expressed ameloblast markers, could be maintained for more than five months (11 passages) in vitro in media containing modulators of Wnt, Egf, Bmp, Fgf and Notch signaling pathways, and were amenable to cryostorage. When transplanted underneath murine kidney capsules, organoids produced OHAp crystallites similar in composition, size, and shape to mineralized dental tissues, including some enamel-like elongated crystals. DEOs are thus a powerful in vitro model to study mineralization process by dental epithelium, which can pave the way to understanding amelogenesis and developing regenerative therapy of enamel.

## Introduction

Ameloblasts are cells derived from the dental epithelium that direct the highly oriented growth of hydroxylapatite crystallites during tooth development. In all human teeth, as well as the molars of mice, ameloblasts are lost during tooth eruption. Therefore, adult human teeth and mouse molars cannot regenerate enamel beyond superficial remineralization from saliva^1^. In contrast, mouse incisors generate functional ameloblasts throughout life to produce enamel to support the continuously growing teeth. This ameloblast population originates from adult dental epithelial stem cells (aDESCs), which reside in a niche called the labial cervical loop^2^.

To facilitate the investigation of this unique stem cell population, two-dimensional or three-dimensional culture systems for aDESCs isolated from the incisors of adult mice have been established^3–5^. Hermans et al. recently established tooth organoids using early-postnatal mouse molar and incisor^6^. As human, aDESCs are not readily available, and recent reports suggested to induce aDESC from human induced pluripotent stem cells (hiPSCs) and dental follicle cells from unerupted wisdom teeth have been used^7–9^. Some of the reports showed ability to secrete enamel matrix proteins^6,9^ and even deposit biomineral^7,8^ when epithelial cells were combined with mesenchyme. However, the function of ameloblasts in the absence of mesenchyme has not been studied, and the structure of crystallites in organoids has not yet been been characterized.

Knowledge about the signaling pathways regulating the fate of stem cells is essential to establish organoids cultures. Molecular developmental studies have shown that numerus pathways are involved in the regulation of dental stem cells during early stage of tooth development. Sonic hedgehog (Shh) and fibroblast growth factor (Fgf) signaling are essential for initiation of tooth bud^10,11^. Wnt signaling also plays an important role in early tooth morphogenesis^12,13^. Wnts, Shh, bone morphogenetic proteins (Bmps) and Fgfs expressed from the primary enamel knot promote expansion and elongation of epithelial tissue into dental cusps^14^. During cap and bell stage, mesenchymal Fgf10 maintains the survival of epithelial progenitors, and the spatiotemporal expression of Fgfs regulates proliferation and invagination of dental epithelial cells^15^. Notch signaling regulates the fate and differentiation of dental epithelial stem cells by interacting with Fgf signaling^16^.

In this study, we established long-term culture conditions for dental epithelial organoids (DEOs) generated from murine aDESCs that expressed ameloblast markers when treated with a combination of Wnt, Bmp, Fgf, and Notch signaling modulators. Remarkably, when the organoids were transplanted underneath the murine kidney capsule, they not only secreted enamel matrix proteins but also deposited hydroxylapatite (OHAp) biomineral.

## Results

### Murine DEOs originating from single aDESCs

**Fig. 1:**
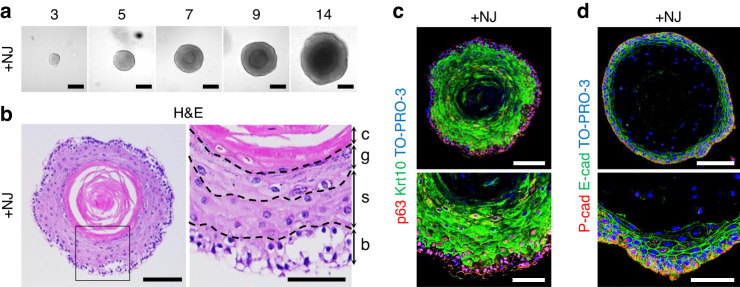
Establishment of a 3D culture system for murine dental epithelial organoids. **a** Differential interference contrast (DIC) images of dental epithelial organoids (DEOs) on days 3, 5, 7, 9, and 14 of culture from adult dental epithelial stem cells (aDESCs) in medium containing Wnt3a, R-spondin1, epidermal growth factor (Egf), and Jagged1 (+NJ). Scale bar = 100 μm. **b–d** DEOs grown in +NJ for 2 weeks. Hematoxylin and eosin (H&E) staining of paraffin sections of a DEO (**c** left, scale bar = 100 μm). On the right is a magnified image of the outlined area showing four epithelial cell layers (**c**, right, scale bar = 50 μm). c cornified, g granular, s spinous, b basal. Confocal image of paraffin sections of a DEO costained for P63 (**d** red), cytokeratin 10 (Krt10, **d**, green), and TO-PRO-3 (**d** blue). Scale bar = 100 μm (**d** top). Higher magnification (**d** bottom, scale bar = 50 μm). Confocal image of paraffin sections of a DEO costained for P-cadherin (**e**, red), E-cadherin (**e** green), and TO-PRO-3 (**e** blue). Scale bar = 100 μm (**e**, top). Higher magnification (**e**, bottom, scale bar = 50 μm)

We isolated aDESCs from extracted apical buds of wild-type C57BL/6 mouse incisors by collagenase, dispase and trypsin-EDTA treatment and suspended the cells in Matrigel. First, we grew aDESCs in culture media used for taste bud organoids^17^, which contains essential factors for the culture of epithelial organoids, (Wnt3a, R-spondin1 and epidermal growth factor; Minimal in Table S1)^18^ with Noggin and Jagged1 (+NJ in Table S1). In this medium, aDESCs grew as organoids with a sphere appearance displaying a multilayered inner structure (Fig. 1a). Sectioning and histological staining by hematoxylin and eosin (H&E) staining revealed that the DEOs retained a stratified epithelial structure (Fig. 1b), a typical multilayered structure found in skin epidermis^19^. The epithelial basal cell markers, p63 and P-cadherin^20–22^ were specifically expressed in the outermost layers of the organoids (Fig. 1c, d). The expression of cytokeratin (Krt) 10, a marker of spinous layers^19^, was completely exclusive to p63 (Fig. 1c). E-cadherin was partially expressed compared to Krt10 in the suprabasal layers of the organoids (Fig. 1c, d).

### Fibroblast growth factor 10 induced bud formation of organoids

**Fig. 2:**
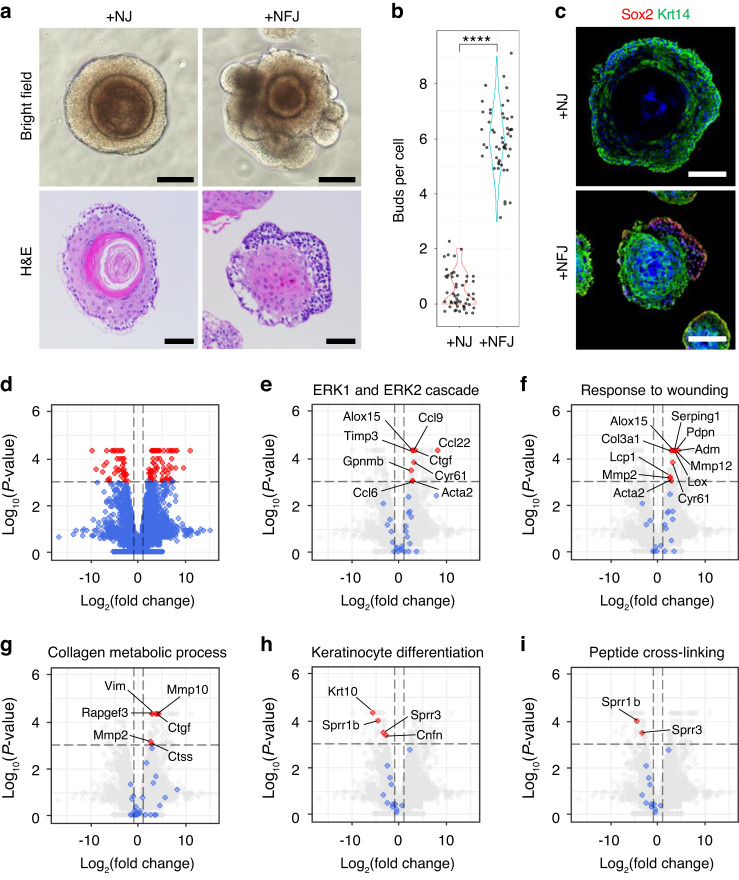
Effect of fibroblast growth factor 10 (FGF10) treatment on DEOs. **a**–**c** DEOs were grown in +NJ or FGF10-supplemented medium (+NFJ) for 2 weeks. Bright-field images of DEOs (**a**, top, scale bar = 50 μm). H&E staining of paraffin sections of DEOs (**a**, bottom, scale bar = 50 μm). Number of buds per DEOs (**b**, *n* = 50). Confocal image of paraffin sections of DEOs costained for Sox2 (**c** red), cytokeratin 14 (Krt14, **c** green), and TO-PRO-3 (**c** blue). Scale bar = 100 μm (**c**). Volcano plots of differentially expressed genes (DEGs) of DEOs grown in +NFJ compared with those of DEOs grown in +NJ. Fold changes are shown for all DEGs (**d**) or for genes related to specific ontology (GO) terms (**e**–**i**) as red (|fold change| > 2 and *P*-value < 0.001), blue (|fold change| < 2 or *P*-value > 0.001), and gray (not related to the indicated GO term) dots. Vertical dashed lines indicate −2-fold and +2-fold changes. A horizontal dashed line indicates a *P*-value of 0.001

Fgf signaling is essential for the initiation and invagination of dental epithelium during early tooth development^23^. Among the various isotypes of Fgf, Fgf10 is expressed in the mesenchyme of developing incisors and plays important roles in cervical loop formation and maintenance^15^. To validate the capacity of DEOs to differentiate into dental epithelium, we added FGF10 to the culture media (+NFJ in Table S1). Notably, the formation of buds from DEOs was observed in +NFJ (Fig. 2a, upper panels and Fig. 2b). Histological analysis showed suppression of keratinocyte differentiation in the core of organoids grown in +NFJ (Fig. 2a, lower panels). Krt14, a marker of epithelial basal cells^24^, was expressed in both organoids grown with and without FGF10 (Fig. 2c, green). However, the expression of Sox2, a dental stem cell marker^25^, was observed only in budding regions of organoids grown in +NFJ (Fig. 2c). Differentially expressed gene (DEG) analysis based on RNA-seq results of the organoids grown with or without FGF10 showed that the transcription of 907 genes was significantly changed by FGF10 treatment (*P* < 0.05, Fig. 2d). For further analysis, upregulated (602) and downregulated (305) genes were subjected to gene ontology (GO) analysis. Upregulated and downregulated genes were significantly matched with 554 and 284 biological process (BP) terms, respectively, and the top 10 results of each group were visualized (Fig. S1a). The results revealed that the genes related to extracellular structure and matrix organization and cell adhesion and migration were upregulated, and the genes related with epidermis development were downregulated in organoids by FGF10 treatment (Fig. S1a). To visualize genes related to the GO terms, we annotated the volcano plot with the name of genes belonging to each term (Fig. S1b and Fig. 2e–i). FGF10 significantly increased the expression of downstream and upstream genes of extracellular signal-regulated kinases (ERKs, Fig. 2e), and extracellular matrix (ECM)-related genes including collagen (Col3a1, Fig. 2f) and matrix metalloproteinases (Mmp 2, Mmp10, Mmp12, Fig. 2f, g). In contrast, small proline rich proteins (Sprr1b, Sprr3) and cornifelin (Cnfn), the genes involved in the keratinization process^19^, and Krt10 were significantly decreased by FGF10 treatment (Fig. 2h, i). Gene set enrichment analyses (GSEA) of significantly regulated genes in +NF compared to +N showed the positive enrichment of cell cycle- related GO terms and negative enrichment of skin development-related GO term (Fig. S2).

### Depletion of Jagged1 aided bud formation of organoids

Notch signaling plays a major role in epidermis development and homeostasis. Activation of Notch signaling by Notch ligands including Jagged1, suppresses the basal and cornified layer and promotes spinous and granular layer development in stratified epithelium^26^. DEG analysis of RNA-seq results of organoids grown in +N and +NJ medium showed that stem cell- (Bmi1, Sox2, and Gli1), proliferation- (Mki67, Pcna, and Ccnb1), and ameloblast-related markers (Ambn and Amelx) were not significantly regulated in +NJ compared to +N. However, keratinocyte related markers (Cnfn, Sppr2k, and Krt10) showed significant upregulation in +NJ compared to +N (Fig. S3).

**Fig. 3:**
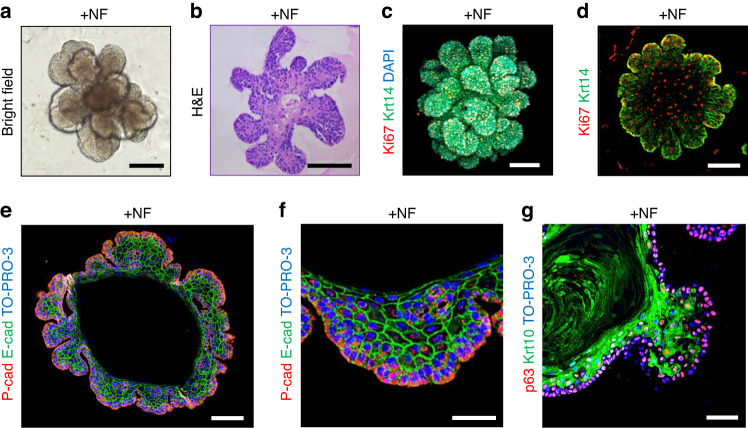
DEOs cultured in Jagged1-depleted, FGF10-supplemented medium form robust basal epithelial layers. **a**–**g** DEOs were grown in Jagged1-depleted, FGF10-supplemented medium (+NF) for 2 weeks. Bright-field image of a DEO (**a**, scale bar = 100 μm). H&E staining of paraffin sections of a DEO (**b**, scale bar = 100 μm). Maximum intensity projection of serial Z-section confocal images of a whole-mount DEO stained for Ki67 (**c** red), Krt14 (**c** green), and DAPI (**c** blue, scale bar = 50 μm). Confocal image of paraffin sections of a DEO costained for Ki67 (**d**, red) and Krt14 (**d**, green, scale bar = 50 μm). Confocal image of paraffin sections of a DEO costained for P-cadherin (P-cad, **e** red), E-cadherin (E-cad, **e**, green), and TO-PRO-3 (**e**, blue, scale bar = 50 μm). Higher magnification (**f** scale bar = 25 μm). Confocal image of paraffin section of a DEO costained for P63 (**g** red), Krt10 (**g** green), and TO-PRO-3 (**g** blue, scale bar = 25 μm)

Active bud formation was observed in DEOs grown in FGF10-supplemented and Jagged1-depleted culture media (+NF in Table S1, Fig. 3a–g). The buds consisted of basal and suprabasal layers reminiscent of an invagination of epithelium during development of epidermal appendages, in which E-cadherin-positive suprabasal cells provides centripetal force to make bud structure^27^. Whole mount (Fig. 3a) and section (Fig. 3d) staining of DEOs grown in +NF showed that most cells expressed Ki67, a proliferation marker, whereas, of Krt14 expression was dominant at the verge of buds. The exclusive staining patterns of E-cadherin and P-cadherin (Fig. 3e, f), and Krt10 and p63 (Fig. 3g) revealed well-developed basal and suprabasal layers in the buds.

**Fig. 4:**
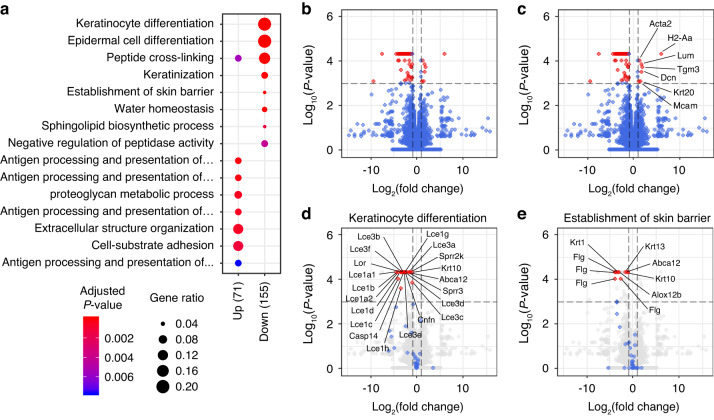
Transcriptomic change of DEOs cultured in Jagged1-depleted, FGF10-supplemented medium. **a** A dot plot of GO overrepresentation results for upregulated (Up) and downregulated (Down) DEGs in DEOs grown in +NF compared with DEOs grown in +NJ. The color and size of the dots indicate the *P*-value and gene ratio of each representative GO term. Volcano plots of DEGs of DEOs grown in +NF compared with DEOs grown in +NJ. Total (**b** and **c**) or indicated GO term-related (**d**, **e**) genes are displayed as red (|fold change| > 2 and *P*-value < 0.001), blue (|fold change| < 2 or *P*-value > 0.001), and gray (not related to the indicated GO term) dots. Vertical dashed lines indicate −2-fold and +2-fold changes. A horizontal dashed line indicates a *P*-value of 0.001

GO analysis based on DEG (+NJ vs. +NF, *P* < 0.05) identified 71 and 155 BP terms for the upregulated and downregulated gene groups (Fig. 4a). The top GO terms were similar to those of +NFJ; extracellular structure organization and cell-substrate adhesion were annotated for the upregulated gene group, whereas epidermal cell differentiation and keratinization-related terms were annotated for the downregulated gene group (Fig. 4a). Eighty-one genes were significantly changed in organoids grown in +NF compared with +NJ (|fold change| > 2 and *P*-value < 0.01, Fig. 4b, red dots). Genes related to cell motility (α-actin2, Acta2), cell adhesion (melanoma cell adhesion molecule, Mcam), and extracellular matrix (derocin, Dcn; lumican, Lum) were included in 7 upregulated genes (Fig. 4c). Among 74 downregulated genes, various keratinocyte differentiation and keratinization-related genes were found (ATP-binding cassette sub-family A member 12, Abca12; arachidonate lipoxygenase 3, Alox12b; caspase 14, Casp14; Cnfn; profilaggrin, Flg; late cornified envelope, Lce1a1/1a2/1b/1c/1d/1g/3a/3b/3c/3d/3e/3f; loricrin, Lor; Sprr2k, Fig. 4d, e). In addition, downregulation of Krt1/10/13 and upregulation of Krt20 were observed (Fig. 4c–e).

To clarify the effect of Fgf10, GO analysis was performed using commonly upregulated (Fig. S4a) and downregulated (Fig. S4b) genes in +NF and +NFJ compared to +N. Various metabolic process-related GO terms were enriched in commonly upregulated genes (Fig. S4c). Interestingly, the GO terms enriched in commonly downregulated genes were mainly related to skin development (Fig. S4d).

### Inhibition of Notch signaling boosted the basal layer of organoids

**Fig. 5:**
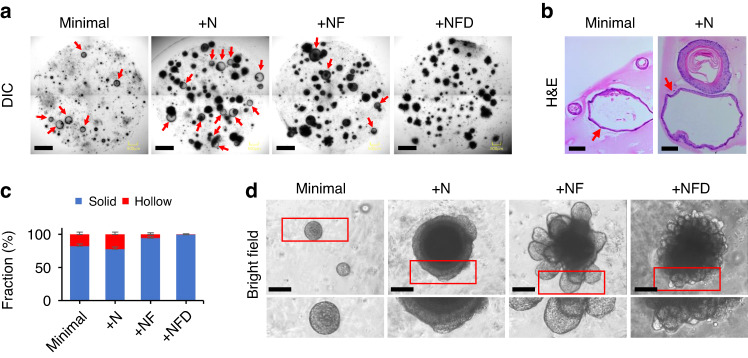
Morphology of organoids grown at different media compositions. Organoids were grown from aDESCs embedded in Matrigel using media supplemented with Wnt3a, R-spondin1, and Egf (minimal), minimal + Noggin (+N), +N + Fgf10 (+NF), or +NF + dibenzazepine (+NFD) for 2 weeks. **a** Maximum intensity projection of serial Z-section DIC images of whole well with multiple organoids. Scale bar = 500 µm. **b** H&E-stained paraffin sections. In **a** and **b**, red arrows indicate hollow spheroids. Scale bar = 100 µm. **c** Bar plot of the fraction of solid (blue) and hollow (red) spheroids as a function of the media composition (*n* = 3). Data are represented as the mean ± SD. **d** Bright-field images of representative organoids. Scale bar = 100 µm

To find optimal conditions for organoid growth, we examined various combinations of growth factors. While dissociated single cells from the dental epithelium initially formed small organoids in the minimal medium, they either ceased growing (Fig. 5a and Fig. S5a, b) or formed hollow spheres containing only a single-cell layer, indicating loss of stemness to maintain epithelial structure (Fig. 5a–c, red arrows). We then added Noggin. When media were supplemented with Noggin (+N in Table S1 and Fig. 5a, b), which expands intestinal epithelial stem cells in both mouse and organoids^28,29^, organoids grew exponentially, but they still formed hollow spheres (Fig. 5a–c, red arrows). Furthermore, we examined dibenzazepine (DBZ; NFD in Table S1), a Notch inhibitor^30^, since genetic or chemical inhibition of Notch signaling increases the proportion of basal stem cells in epidermal tissues^26^. The growth rate of organoids cultured in +NF or +NFD media was similar to that of organoids cultured in +N medium (Fig. 5a, b). However, the fraction of hollow spheroids was reduced significantly when organoids were cultured in +NF or +NFD media (Fig. 5a, c).

The aDESCs cultured in +N medium formed spherical organoids with multilayered structures (Fig. 5d). FGF10 treatment induced budding structures (+NF, Fig. 5d) that contained epithelial stem cells and their descendants^18^. Interestingly, in +NFD medium (Fig. 5d), organoids formed smaller, but more numerous buds compared to those cultured in +NF medium. The morphological differences between organoids cultured in +NF and +NFD media were confirmed by serial Z-stack confocal images (Fig. S6a).

**Fig. 6:**
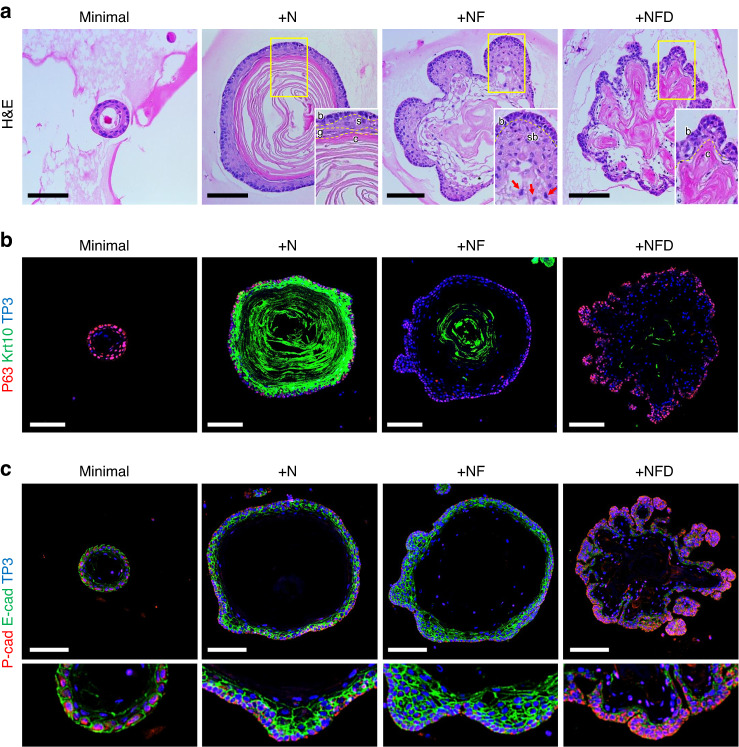
Histological and immunohistological analysis of DEOs. Organoids were grown from aDESCs embedded in Matrigel using media supplemented with minimal, +N, +NF, or +NFD for 2 weeks. **a** H&E-stained paraffin section. Scale bar = 100 µm. Confocal images of paraffin sections immuno-labeled for P63 (**b** red) and Krt10 (**b** green), or P-cadherin (P-cad, **c** red) and E-cadherin (E-cad, **c** green), and counterstained for nuclei with TO-PRO-3 (TP3, blue). Scale bar = 100 µm

H&E staining of sections of organoids grown in +N media revealed well-developed basal (b), spinous (s), granular (g), and cornified (c) layers (Fig. 6a). This is typical for the stratified structure of skin rather than dental epithelium. Dominant expression of Krt10, assessed by immunofluorescence microscopy, is consistent with a multilayered differentiating epithelium (Fig. 6b).

In +NF medium, organoids lost their stratified epithelial layers and generated budding structures with expansion of a single layer of columnar basal cells (b) and a multi-layer of flattened suprabasal cells (sb, Fig. 6a). The invasion of epithelial organoids into the Matrigel is reminiscent of epithelial invagination into mesenchyme, an early event during tooth development that requires E-cadherin expression in suprabasal cells^10,31^. Indeed, organoids cultured in +NF medium showed enrichment of E-cadherin-positive cells in the suprabasal layer (Fig. 6c and Fig. S6b). With its distinct star-shaped cells (Fig. 6a, +NF, red arrows), the suprabasal layer in the organoids resembled the stratum intermedium of the tooth bud at bell stage.

Basal (b) and cornified (c) layers of organoids were well-developed in the +NFD medium (Fig. 6a). In these organoids the basal layer was dominant, as indicated by strong expression of P63 (Fig. 6b), a marker of epithelial stem cells^32^. The P-cadherin, which dominantly expresses in the inner enamel epithelium of tooth bud in the early- to mid-bell stage^33^ and a marker of transient amplifying population of the mouse incisor epithelium^34^, also highly expressed in the organoids grown in +NFD medium (Fig. 6c). However, E-cadherin-positive suprabasal cells were rarely observed in the small bubble-like structures of the organoids cultured in +NFD medium (Fig. 6c and Fig. S6c). The lack of suprabasal cells resulted in the loss of centripetal force to invaginate into Matrigel and bubble-like structures rather than budding structures observed in organoids grown in +NF medium. Inhibition of Notch signaling with simultaneous activation of Fgf signaling resulted in a significant expansion of basal layer, which indicates +NFD medium as the most favorable condition to maintain the aDESCs (Fig. 6c).

Interestingly organoids grown in both +N and +NFD medium contained eosinophilic cores (Fig. 6a, +N and +NFD), however, the antigenicity of the cores were different; the cores of organoids grown in +NFD medium showed low antigenicity for Krt10 antibody (Fig. 6b, +NFD). The DEG result of +NJ, +ND, +NFJ, +NFD compared to +N revealed that supplement of Jagged1 significantly increased expression of Krts significantly. However, supplement of FGF10, DBZ, or both significantly decreased the expression of Krts including Krt10 (Fig. S7). These results indicate that the eosinophilic cores observed in organoids grown in +NFD medium are different corneous found in skin tissues. In addition, induction of the skin development (GO:0043588) related genes by Jagged1 or DBZ treatment was suppressed by FGF10 supplement, which indicates that the FGF10 inhibits epidermal fate decision of the aDESCs in DEOs (Fig. S8).

### Transcriptomic analysis reveals ameloblast marker expression in organoids cultured in +NFD medium

**Fig. 7:**
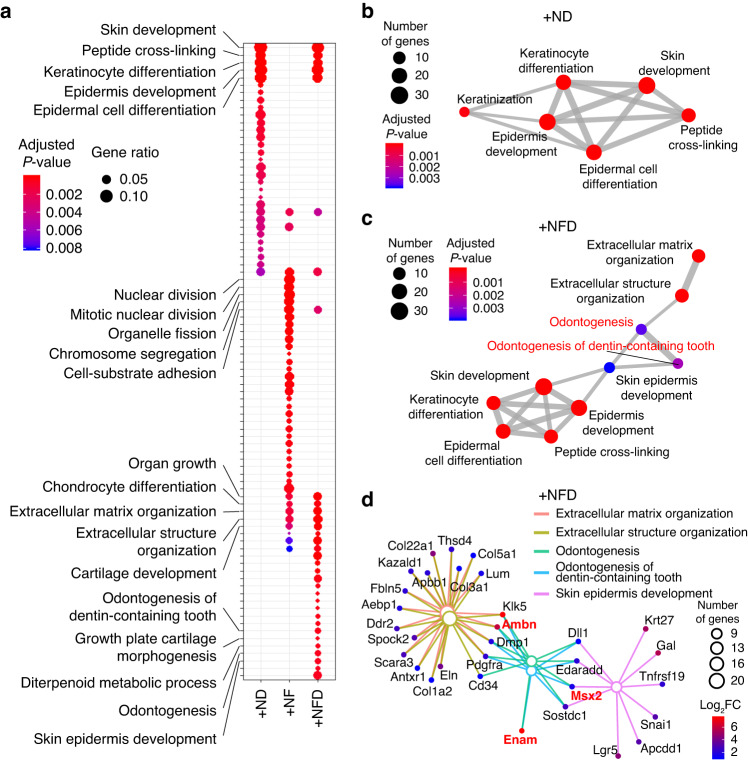
Functional analysis of genes differentially expressed in organoids grown at different media compositions. Analyses for organoids grown in +ND, +NF, and +NFD media were referenced to expression in +N media. **a** Dot plot visualizing gene ontology (GO) overrepresentation results for upregulated genes. Unique GO terms identified for each condition are labeled (a fully labeled plot is provided in Fig. S4a). The color and size of the dots indicate the *P*-value and gene ratio of each representative GO term, respectively. Enrichment maps of skin development-related GO terms for +ND (**b**) and +NFD (**c**) media. The color and size of the dots indicate the *P*-value and gene number of each representative GO term, respectively. The thickness of the solid lines linking the dots represents the number of genes shared between two GO terms. **d** A gene network of extracellular matrix-, odontogenesis-, and skin epidermis development-related GO terms (large dots) and related genes upregulated in the organoids cultured in the +NFD medium. The size of the open dots indicates the number of genes found in each term. Upregulated genes related to each term are visualized as closed dots. The color of the small dots represents the fold change in gene expression

We next compared expression profiles among the organoids grown in different culture conditions by performing bulk RNA-seq. DEGs were identified, and genes that were significantly upregulated in organoids cultured in +ND, +NF, and +NFD compared to those grown in +N medium (*P* < 0.05) were functionally annotated with GO terms. While in +ND medium skin development-related genes were upregulated, cell division- and extracellular matrix organization-related genes predominated in +NF (Fig. S9a and Fig. 7a). There was some overlap of GO terms, such as skin development- or extracellular matrix-related terms, between +NFD and +ND or +NF-grown organoids; however, only organoids cultured in +NFD exhibited upregulation of genes associated with odontogenesis (Fig. 7a).

We produced GO term network maps to visualize relationships between terms based on the number of genes they share^35^. Skin- and keratinization-related terms in +ND formed a closed network, which indicated functional redundancy among the terms (Fig. 7b). Interestingly, in organoids grown in +NFD medium, the closed network extended to include odontogenesis- and extracellular matrix organization-related terms (Fig. 7c). Genes related to ameloblast differentiation (ameloblastin, Ambn; ectodysplasin-A receptor-associated adapter protein, Edaradd; enamelin, Enam; and Msh homeobox 2, Msx2) were found in genes associated with the newly added terms in the network (Fig. 7d). In particular, the genes encoding proteins related to amelogenesis, such as amelogenin (Amelx), Ambn, Enam, Mmp20 and Tmem108, showed relatively high expression in organoids grown in +NFD medium (Fig. 7d and Fig. S9b).

To assess the similarity between organoids cultured in different media and the dental epithelium, we extracted gene sets for nine distinct cell types from a comprehensive single-cell RNA sequencing study^36^. GSEA revealed that pre-ameloblast, proximal ameloblast, and distal ameloblast gene sets were significantly enriched in the genes upregulated in +NFD but not in +ND or +NF media (Fig. S9c).

Based on the combined evidence from histological examination and gene expression patterns, the +NFD medium best supports growth of DEOs from murine aDESCs without loss of their characteristics as the dental epithelium. However, organoids entered culture crisis around passage 5, similar to what was observed in other systems^18^. Therefore, we examined the effects of supplementing +NFD medium with A83-01, an inhibitor of Smad signaling; SB202190, a p38 MAPK inhibitor; and nicotinamide, an amide form of vitamin B3 that increased the number of passages of colon crypt organoids to over twenty^18^. While the organoids grew normally with A83-1 (Fig. S10a), organoid growth was suppressed with SB202190 (Fig. S10a). However, organoids grown with A83-1 and nicotinamide (+NFDNiA) expanded over 5 months and continued to grow actively after being passaged 11 times (Fig. S10b, c). Importantly, single cells isolated from organoids did not lose their organoid-forming capacity even after freezing, storing, and thawing (Figs. S10b–d). This greatly enhances the scope of the organoid culture method we developed herein.

### Generation of crystals and calcified tissues from organoids

**Fig. 8:**
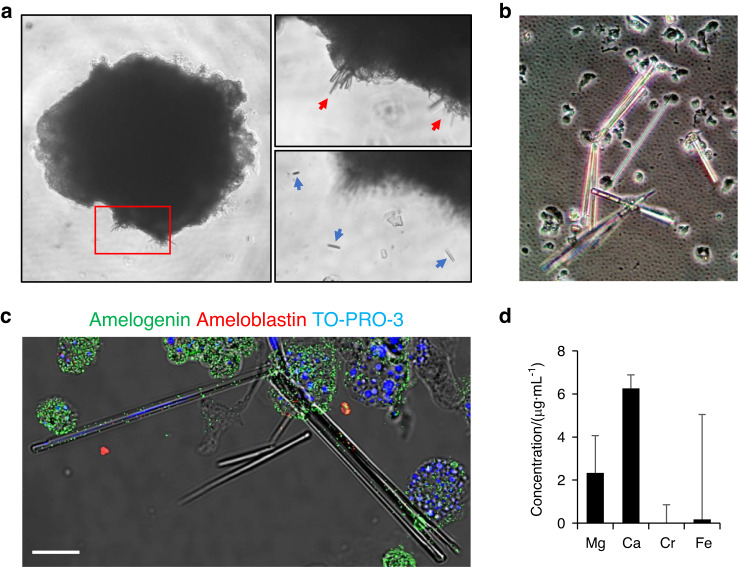
Suspension culture of DEOs. **a**–**d** DEOs grown in Matrigel for 2 weeks with +NFDNiA isolated from the gel and cultured in suspension with additionally supplemented with smoothened agonist (SAG). Bright-field image of a DEO (**a**, left panel) and magnified image of the area indicated by red lined box (**a**, right panels, red and blue arrows indicate crystals growing and being shed from the organoid, respectively). The shed crystals were collected and attached on slide glass for immunohistochemistry (**b** and **c**) or subjected into inductively coupled plasma (ICP) mass spectrometry for analysis of metal ion composition (**d**). Bright image of attached crystals (**b**). Confocal image of crystals costained for amelogenin (**c**, green) and ameloblastin (**c** red). Nucleus were counterstained with TO-PRO-3 (**c**, blue). Scale bar = 20 μm (**c**)

The loss of basement membrane between dental epithelium and mesenchyme prior to enamel matrix secretion and the critical roles of the ECM in ameloblast differentiation have been reported^37^. With the loss of basement membrane, preameloblasts become post-mitotic and responsive to Shh signaling^38^. To examine the effect of ECM and Shh signaling, we isolated DEOs grown in Matrigel for 2 weeks in +NFDNiA and cultured them as suspended on ultra-low attachment plates with media additionally supplemented with smoothened agonist (SAG), an agonist of Shh signaling. After 2-week suspension culture, formation of crystals from the surface of organoids was observed (Fig. 8a, red arrows). Some crystals were shed into the culture media (Fig. 8a, blue arrows).

The enamel matrix proteins and their supramolecular structures are essential for control of the organization of apatite crystals in enamel^39^. Especially amelogenin, a most abundant protein in enamel matrix, forms self-assembled spherical structures which aligned with long axes of growing enamel crystallites^40^. To examine whether the enamel matrix proteins were incorporated in the crystals or not, we attached the shed crystals on silane-coated slide glass (Fig. 8b) and perform immunohistochemistry for amelogenin and ameloblastin (Fig. 8c). We observed dots of amelogenin were aligned along the long axes of the crystals (Fig. 8c, green). Rarely found compared with amelogenein, but the ameloblastin also detected on the crystals (Fig. 8c, red). Inductively coupled plasma (ICP) mass spectrometry revealed that calcium (Ca) and magnesium (Mg) were accumulated in the crystal structures (Fig. 8d).

**Fig. 9:**
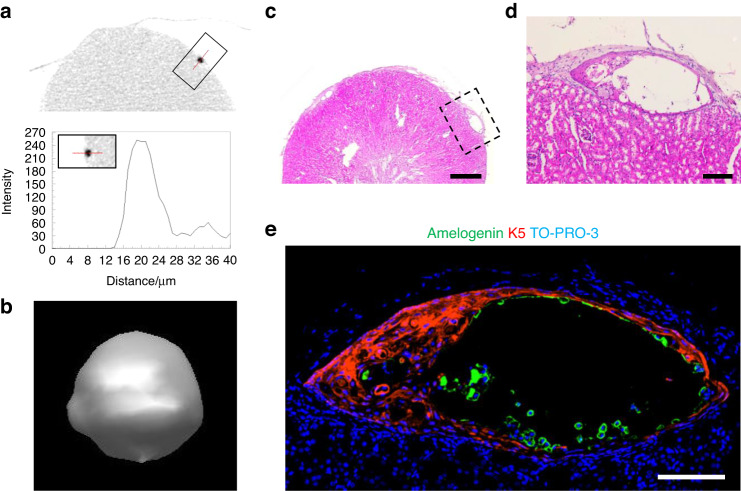
Radiological and histological analysis of DEOs transplanted into kidney. **a**–**e** DEOs were transplanted under kidney capsule and grew for 8 weeks. A cross section (**a**) and reconstructed (**b**) image of micro-computed tomography (micro-CT) of extracted kidney. H&E staining of paraffin sections of decalcified kidney (**c**, **d**). Confocal image of the sections costained for amelogenin (**e**, green) and cytokeratin 5 (K5, **e** red). Nucleus were counterstained with TO-PRO-3 (**e** blue). Scale bar = 500 μm (**c**), 100 μm (**d**, **e**)

Kidney transplantation is a useful method for validating the capacity of tissues to mineralize^41^. To examine amelogenic capacity, we transplanted organoids cultured in +NFDNiA medium under the kidney capsule of mice. Visual inspection of kidneys harvested 8 weeks after transplantation revealed the presence of whitish masses under the capsule (Fig. S11), suggesting the precipitation of a mineral. We found that the organoids transplanted under the kidney capsule generated a radiopaque object (Fig. 9a, b). The object was shown as an empty space surrounded by cells in decalcified section of kidneys (Fig. 9c, d). The enamel matrix proteins guiding mineral deposition during the early stage of amelogenesis are broken down by proteases and peptidases, and they are fully mineralized by mature ameloblasts^1^. In fully mineralized enamel, only ~1–3 percent of proteins remain, and the remaining enamel comprises minerals. As a result, only an empty space is found in the enamel space of the decalcified tooth^42^. The surrounding cells were cytokeratin 5 (K5)-positive (Fig. 9e, red), which confirms the epithelial origin of the cells. Amelogenin was only detected as a thin layer between empty space and surrounding cells or around the cells shed into the empty space (Fig. 9e, green).

**Fig. 10:**
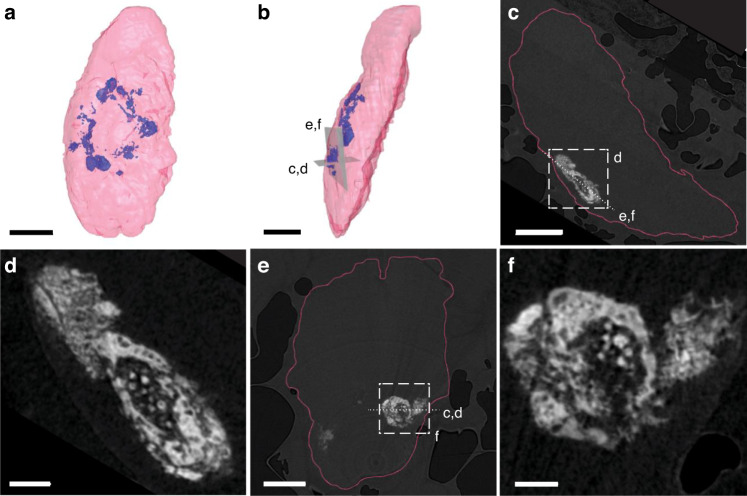
DEOs transplanted into the kidney capsule deposit hydroxylapatite crystals. **a**, **b** Alternate views of a 3D-rendering of the segmented µCT reconstruction of a kidney harvested 8 weeks after transplantation of DEOs cultured in +NFDNiA medium (blue: radiopaque mass; pink: soft tissue). Scale bar = 2 mm. **c** Virtual section oriented as shown in **b** and orthogonal to that shown in (**e**), in the direction indicated by the dotted line. Scale bar = 2 mm. **d** Close-up of the radiopaque mass shown indicated in (**c**). Scale bar = 0.5 mm. **e** Virtual section oriented as shown in **b**, and orthogonal to that shown in **c**, in the direction indicated by the dotted line. Scale bar = 2 mm. **f** Close-up of the mass shown in **e**. In **c**–**f**, the intensity of the gray scale image is proportional to the radio-density and the surface of the kidney is indicated (pink line). Scale bar = 0.5 mm

Micro-computed tomography (μCT, 2 µm isotropic voxel pitch) confirmed the presence of radiopaque masses (Fig. 10a–f). Inspection of slices through the µCT reconstructions (Fig. 10d, f) revealed that the masses were roughly ellipsoidal in shape, where the longest axis reached 1.5 mm or more, and the shortest was typically on the order of 200–350 µm. Masses consisted of irregular aggregates of radiopaque particles. Some of the larger masses contained a shell with a higher density of particles and a core with a lower density.

**Fig. 11:**
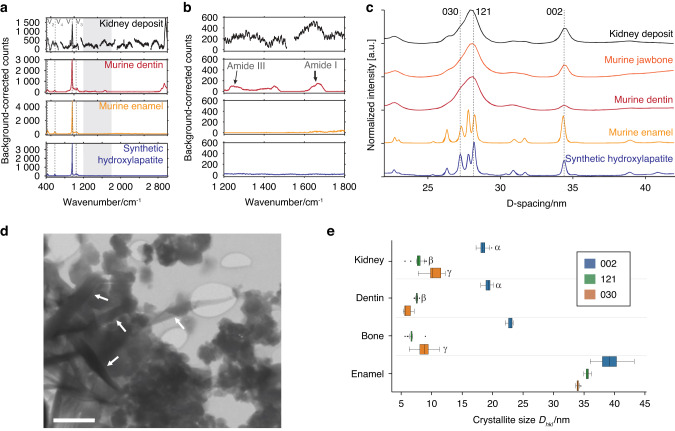
Analysis of material characteristics of deposited biomineral by kidney-transplanted DEOs. **a**–**c** Background-corrected μRaman spectra (**a**, **b**) and μXRD patterns (**c**) of radiopaque masses in the kidney (black), mature murine incisor enamel (yellow), mature murine dentin (red), murine jawbone (orange), and synthetic hydroxylapatite (OHAp, blue). In **a**, the ν_1_, ν_2_, ν_3_ and ν_4_ modes of the phosphate ion are indicated, and the region shown in **b** is highlighted in gray. In **b**, the regions corresponding to Amide I and Amide III bands of proteins are indicated. In **c**, peaks corresponding to the 030, 121, and 002 reflections are indicated. **d** STEM-in-SEM image of mineral aggregates isolated from masses in the kidney, using bright-field contrast. High aspect ratio crystallites are indicated by white arrows. Scale bar = 500 nm. **e** Box plot of the coherently scattering domain size *D*_*hkl*_ (“crystallite size”) normal to the 002, 121, and 030 planes for OHAp deposited in the kidney (*n* = 25 technical replicates), and in murine dentin (*n* = 25), jawbone (*n* = 21), and enamel (*n* = 25). *D*_*hkl*_ were determined by Scherrer analysis (see Fig. S11 and Table S3). Kruskal–Wallis tests were rejected for each direction (*P*-value < 0.05). For each box, the central vertical line indicates the median, and the left and right edges of the box indicate the first and third quartiles. The whiskers represent 1.5 times the interquartile range. Outliers beyond this range are shown as individual points (solid black circle). Except for the groups labeled by identical Greek letters, the means of all the groups were found to differ significantly (Dunn’s test with Bonferroni correction, *P*-value < 0.05)

Elemental composition from energy dispersive X-ray spectroscopy (SEM-EDS) showed that masses excised from the kidney primarily contained calcium, phosphate, and oxygen (Fig. S12) and minor elements (Na, Mg) also found in murine enamel. Signals for elements consistent with proteins or residual organics from surrounding tissue (C, N, S) were also observed in the kidney deposit. A comparison of micro-Raman (µRaman) spectra of kidney deposit, murine dentin, murine enamel, and synthetic OHAp revealed characteristic bands corresponding to the vibrational modes (ν_1-4_) of phosphate in OHAp (Fig. 11a and Table S2), which are distinct from bands in other calcium phosphate minerals (Fig. S13). Vibrational bands corresponding to Amide I and III peaks in proteins were observed in murine dentin and the kidney deposit only (Fig. 11b and Table S2)^43^. Protein bands are commonly observed in dentin^44^, and the kidney deposit was not bleached to remove soft tissue prior to Raman experiments; thus, these peaks are expected. The presence of OHAp in the kidney deposit was confirmed by comparing radially averaged synchrotron X-ray microdiffraction (μXRD) patterns of kidney masses with murine enamel, dentin, and bone and a synthetic OHAp sample (Fig. 11c and, for an example of a 2D pattern, Fig. S14). Taken together, these results provide strong evidence that the radiopaque deposits in the kidney are composed of OHAp, likely interspersed with proteinaceous material. There are no indications that another mineral phase is present, even though the techniques used here may not be sensitive to minor constituents (<5–10 %), especially of poorly crystalline materials.

We note that diffraction patterns of the mineral from the kidney deposits display significantly broadened peaks compared to those of synthetic OHAp or murine enamel and appear more similar to peaks in murine dentin or bone. While several factors can contribute to peak broadening, a likely cause is the physical size of the crystallites^45,46^. Specifically, the broader peaks in patterns of kidney mineral deposits suggest that the crystallites are smaller than those in murine enamel (Fig. 11d, e).

As a lower bound of the physical crystallite size, we extracted the coherently scattering domain size (“crystallite size,” *D*_*hkl*_) normal to the 121, 030, and 002 planes of OHAp in kidney deposits and murine bone, dentin, and enamel using Scherrer analysis of peak widths extracted from diffraction patterns by fitting (Fig. S15 and Tables S3, 4)^47^. Kruskal–Wallis tests indicated that for each of the three directions, the size distributions determined for the four sample types were not identical (Table S5). Inspection of the distributions revealed that, in the kidney deposits and OHAp in murine dentin, bone, and enamel, the mean size in the *c*-axis direction (*D*_002_) was elevated compared to the orthogonal directions (*D*_030_) by a factor of up to approximately three (Fig. 11e), likely reflecting a preference for OHAp crystal growth in the *c*-axis direction. However, the size distributions in the kidney deposits are more like those in dentin than those in enamel (Table S6 for *P*-values for pairwise comparisons using Dunn’s test with Bonferroni correction for multiple comparisons). Specifically, the distributions of *D*_002_ in the kidney and dentin were statistically indistinguishable (Tables S5 and 6S6), as were those for *D*_121_ in the kidney and dentin.

Confirming this assessment, analysis of bleached kidney mineral deposits by electron microscopy revealed numerous irregular aggregates of mineral particles 10 s to 100 s of nm in diameter (Fig. 11d). This is consistent with isolates from bone and dentin in which individual crystallites are typically platelet-shaped (c.a. 50 nm × 25 nm × 4 nm)^48^. However, we also observed acicular structures up to 2 or even 3 µm in length and aspect ratios of 5–10, at lower frequency (Fig. 11d and Fig. S16). These crystallites are highly elongated along the crystallographic *c*-axis with cross-sections approximately 30 nm × 70 nm in size are typically observed in human dental enamel^49^. While it is tempting to conclude that at least some crystallites produced by organoids transplanted in the kidney capsule may thus grow under conditions that resemble amelogenesis, we found these acicular deposits highly sensitive to the beam and have so far been unable to record electron diffraction patterns or lattice images. Whether they are comprised of OHAp, and are indeed elongated along the *c*-axis, thus remains to be seen. However, we are confident in our assessment that the vast majority of the kidney deposits are comprised of nano-crystalline OHAp.

## Discussion

In this study, we report that activation of Fgf signaling and suppression of Notch signaling are required to generate DEOs expressing ameloblast markers. The requirement of FGF10 is expected, because failure of cervical loop development was observed in Fgf10-null mice incisor, and treatment with the recombinant FGF10 protein rescued the cervical loop from apoptosis^15^. In another paper, Harada et al. showed that Fgf10 stimulates the division of progenitor cells^16^, which coincides with our observations in organoids (Fig. 4a, Gene ontology terms like nuclear division [GO:0000280], organelle fission [GO:0048285], and chromosome segregation [GO:0007059] are enriched in FGF10-supplemented conditions). However, the experiments using dental epithelial cells and mice revealed that the activation of the Notch pathway is required for ameloblast differentiation^50–52^.

We suggest that, however, consideration for the experimental context is required in this case. The DESCs isolated from apical buds of mouse incisor successfully generated organoids in initial media condition (+NJ), but the organoids showed stratified epithelial structures with the histological structure of epidermis, not dental epithelium. We found that Jagged1 promotes expression of keratinocyte differentiation-related gene. However, stratified epithelial structures still existed in organoids grown in Jagged1-depleted medium (+N). This indicates that the DESCs of incisor are pluripotent stem cells closer to surface ectoderm stem cells or oral epithelial stem cells, rather than dental tissue specific stem cells or progenitors in hierarchical model for ectodermal organ stem cells^53^. The enamel-to-hair lineage conversion observed in Med1-deficient mice also provides evidence for multipotency of DESCs in incisor^54^. In addition, in vitro culture condition forces the DESCs to skin, rather than dental tissue.

During embryonic epidermis development, BMP and Notch pathway activation induce stratified epidermis. Meanwhile, activation of Wnt signaling with Fgf and inhibitor of BMP from underlying mesenchyme instruct the epithelial cells to make appendages^55^. RNA-seq analysis confirmed that Krt expression increased and decreased according to Jagged1 and DBZ treatment, respectively. Moreover, Fgf10 treatment with DBZ completely suppressed Krt expression. Taken together, in vitro culture condition of DESCs forces the cells to lose their identity and become skin-like structures, and Fgf10 and DBZ treatment keep the identity by preventing keratinocyte differentiation rather than promoting ameloblast differentiation.

We were interested to determine if we could recapitulate in vivo histological structures in the organoids. Li et al. described that E-cadherin-positive suprabasal cells are required for the invagination of epithelium into mesenchyme^10^. Mechanical cell-cell coupling via E-cadherin junction provides centripetal force to make the bud structure^27^. We could observe E-cadherin-positive suprabasal cell layers in the buds of Fgf10-treated organoids, which showed a similar morphological pattern with the early dental epithelial bud of E12.5 mouse embryo. However, we didn’t observe the typical morphology of fully differentiated ameloblasts in our organoids, even though we found biomineral disposition by kidney-transplanted organoids. The loss of histological structures is possibly due to the absence of appropriate mesenchyme, i.e., dental mesenchyme. We tried to recombine our epithelial organoids with a spheroid of various dental mesenchymal cells or dental mesenchyme from mouse embryo development stages (at the bud, cap, and bell stage). Still, we couldn’t observe interactions between the epithelial organoids and the mesenchyme tissue, likely due to the mismatches of developmental time schedules between artificial organoids and embryonic dental mesenchymal tissue. Finding the most appropriate mesenchyme counterpart will be an important topic for future study.

In previous studies, dental epithelial cell-derived organoids showed expression of various marker genes of ameloblast from early markers like E-cadherin, β-catenin, integrin β4, P63, Sox2 and Krt14^4,5^ to late markers such as ODAM, Enamelin, Amelogenin and Ameloblastin^6–9^. Expression of these markers were observed in the DEOs of this study and of note, the enamel matrix proteins were found in the secreted materials from suspension-cultured and kidney-transplanted organoids. Furthermore, the DEOs expressed the late ameloblast markers and generated calcified biomaterials without the aid of mesenchyme in a defined culture condition.

In conclusion, we have established a long-term DEO culture system using aDESCs isolated from the apical bud of mouse incisor. Single cells derived from DEOs retain their organoid-forming capacity, even after freezing, storing, and thawing. In a kidney transplantation model, DEOs expressed Amelx and precipitated significant amounts of OHAp in the extracellular space. While most OHAp crystallites were more similar to crystallites found in bone or dentin compared with those in enamel, some acicular deposits with a high aspect ratio and larger overall size reminiscent of crystallites in enamel were also present. We anticipate that this approach will serve as a model for early amelogenesis and may ultimately enable the growth of fully mineralized enamel.

## Materials and methods

### Single-cell isolation from tissue and organoids for subculture and storage

Dental epithelial tissue of apical mouse incisor buds was extracted from 9-week-old female C57BL/6J mouse as previously described^56^. Collected epithelial tissues were immersed in dissociation solution (a 1:1:1 mixture of TrypLE, 2.2 U·mL^–1^ Dispase II, and 1 mg·mL^–1^ collagenase IV) and placed on ice. Using a Pasteur pipette drawn into a capillary as previously described^57^, tissues were triturated by repeatedly aspirating and expelling the solution and incubated at 37 °C water bath for 30 min. Then equal volumes of chilled basal media (DMEM/F12 containing 2% [v/v] B27, 1% [v/v] N2, and 1% [v/v] penicillin–streptomycin) were added to the solution. The solution was passed through a 40-μm cell strainer and centrifuged at 400 × *g* for 5 min. The supernatant was discarded, and the pellet was suspended in pre-melted Matrigel. The 20 μL of Matrigel containing 5 × 10^3^ cells was seeded per a well of pre-warmed 24-well culture plates and solidified in 37 °C incubator for 10 min before media overlay. All media were supplemented with 1 mmol·L^−1^ *N*-acetylcysteine, 10 μmol·L^−1^ Y27632, 50% (v/v) Wnt3a-conditioned media (CM), 5% (v/v) R-spondin1-CM, and 100 μg·mL^−1^ EGF (minimal medium). Some media were supplemented with one or more of the following: 200 μg·mL^−1^ Noggin (N), 100 μg·mL^−1^ FGF10 (F), 10 mmol·L^−1^ DBZ (D), 0.5 μmol·L^−1^ A83-01 (A), and/or 10 mmol·L^−1^ nicotinamide (Ni). Subculture was performed every 14 days. For subculture, Matrigel was disrupted by pipetting with chilled basal media and organoids were pelleted at 200 × *g* for 5 min. The pellet was resuspended and triturated in pre-warmed TrypLE by pipetting through a pipette as described before. The suspended pieces of organoids were incubated in the dissociation solution at 37 °C for 10 min. The dissociated cells were filtered and embedded into Matrigel in same manner as described above. For long-term storage, the dissociated single cells were frozen and thawed as previously described^57^.

### Kidney transplantation of organoids

Organoids grown in +NFDNiA medium for 14 days were released from Matrigel as described above. Culture was continued in suspension with +NFDNiA medium on a 24-well ultra-low attachment plate (Cell Floater Plates, SPL) for 4 weeks. Organoids with a diameter of 700–800 μm diameter were transplanted under the renal capsules of athymic nude mice (Koat:Athymic NCr-nu/nu, Koatech, Seoul, Korea). Surgery was performed as described previously^41^, using carbon dioxide anesthesia. Six organoids per kidney were transplanted and harvested after 8 weeks. All animal experiments were performed according to the guidelines of the Yonsei University Health System, Intramural Animal Care and Use Committee (YUHS-IACUC). YUHS-IACUC complies with the Guide for the care and use of laboratory animals (National Research Council, USA). The animal study plan was reviewed and approved by this committee (2018-0295).

### Histological and immunohistological analysis

For paraffin sectioning, organoids in their Matrigel matrix were fixed in 4% at room temperature (RT) for 1 h, then cast into Histogel (Thermo Scientific) as per the manufacturer’s instructions. Histogel blocks were fixed in 10% neutral buffered formalin solution overnight. Harvested kidneys were fixed in 4% paraformaldehyde overnight at 4 °C. Fixed kidneys were then immersed in 15 ml 10% EDTA for a month at 4 °C for decalcification. The EDTA solution was changed every week. Kidneys were then embedded in paraffin and sectioned to 4 μm thickness. After deparaffinization, sections were stained using hematoxylin and eosin for histological analysis or subjected to antigen retrieval in citrate buffer pH 6.0 at 121 °C for 15 min for immunohistological analysis. After cooling to RT, sections were treated with blocking solution (1% BSA and 1% goat serum in PBS) and incubated with primary antibodies (see REAGENT or RESOURCE table in Materials section) at 4 °C overnight. Sections were washed and incubated with secondary antibodies (see REAGENT or RESOURCE table in Materials section) and counterstained with TO-PRO-3 (Thermo Scientific). Sections were mounted using PermaFluor (Thermo Scientific) and images were taken on an LSM 700 confocal microscope system (Zeiss).

### Whole-mount staining

Whole-mount staining of organoids was performed as previously described^57^. Images were taken on an MP7 multiphoton microscope system (Zeiss).

### RNA-Seq

A library was constructed using TruSeq RNA Library Prep Kit (Illumina) from total RNA and sequenced on an Illumina HiSeq 2000 sequencer. Reads qualified and pre-processed using FastQC (v0.11.9) and Trim Galore (v0.6.5) were aligned on a reference genome (GRCm38) using TopHat (v2.1.1). Abundance of transcripts was estimated from aligned reads using Cufflinks (v2.2.1). Differentially expressed genes (DEGs) were identified using DEGseq (v1.13.1). GO analysis and GSEA were performed using clusterProfiler (v3.15.1). For enrichment analysis of dental epithelial cell markers, pre-ranked GSEA was performed using gene sets established based on the markers suggested in a comprehensive single-cell RNA sequencing study on the epithelium of adult mouse incisor^36^. Top 100 markers by enrichment score of each cell type were used to compose the gene sets. Data analysis and visualization was performed using R (v3.6), RStudio (v 1.2.5033-1), and Bioconductor (v3.9).

### Size and bud number quantification of dental epithelial organoids

For each Matrigel containing organoids, a series of 6.7 mm × 6.7 mm 50 bright-field images were taken at 100 μm steps in the z-axis direction, using a CQ1 confocal quantitative image cytometer (YOKOGAWA, Japan), and merged into a maximum intensity projection image. Further image processing and measurement of the cross-sectional area of all organoids within the field of view were performed using FIJI^58^. The images were converted into 8-bit black and white images and then the area of particles larger than 50 μm^2^ with circularity between 0.5 and 1 was recorded. To count bud numbers, bright filed images of organoids were collected and number of buds in each organoid was counted (*n* = 50). The distributions of the cross-sectional area were visualized as violin plots using the ggplot2 package in RStudio.

### Statistical analysis

For the comparison of the fraction of hollow spheroids in total number of spheroids grown observed in different growth media, an unpaired, two-tailed Student’s *t* test was used to calculate the *P*-value. Error bars are presented as mean ± standard deviation unless otherwise specified. For DEG analysis, GO analysis, and GSEA, implemented statistical methods in DEGseq and clusterProfiler were used and significance was assessed using *P*-values adjusted using the Benjamini and Hochberg method.

### Sample preparation for mineral analysis

Whole kidneys were stored in 70 % aqueous ethanol at 4 °C until dissection. Mineral aggregates (~1 mm diameter) were excised with a #10 scalpel blade and placed on carbon tape for micro-Raman (μRaman) spectroscopy and scanning electron microscopy energy dispersive spectroscopy (SEM-EDS). The mineral was removed from the tape and fixed between two pieces of Kapton tape for microbeam diffraction (μXRD) mapping.

For STEM-in-SEM imaging, the mineral was bleached with two drops (~50 μL) of 8.25% hypochlorite for 5 minutes at RT, at which time the solution stopped foaming. The sample was then sonicated for 5 minutes in room temperature tap water and sedimented by centrifugation (9 400 × *g* for 2 min). The supernatant was removed, and the pellet was resuspended in 100% ethanol (100 μL). One 50 µL aliquot of this suspension was bleached two additional times. The triply bleached pellet was resuspended in 100 μL of 100% ethanol by briefly vortexing the solution. Lacey carbon grids were treated in a Pelco easiGlow plasma cleaner (Ted Pella, Redding, CA) at 0.39 mbar for 15–30 s. Immediately following, 10 μL of the mineral suspension was drop cast onto the grids and allowed to dry in air. Grids were stored in a dry box at room temperature before being imaged.

Murine dental tissues were collected from wild-type mice at least eight weeks old following intracardial perfusion fixation with phosphate-buffered saline (PBS, approx. 10 mL) followed by 4% (w/v) PFA in PBS (approx. 10 mL). A #10 scalpel blade was used to remove soft tissue, and the hard tissues were dehydrated in an ethanol series at room temperature (30%, 50%, 70%, 90%, 100% aqueous ethanol). For μRaman and SEM-EDS, incisors were extracted, embedded in epoxy resin, and left to cure overnight at RT. Longitudinal (approximately sagittal) sections were ground with increasingly smaller grit size (SiC paper, 600–1 200 grit) and polished using water-based polycrystalline diamond suspensions (3 µm, 1 μm).

For μXRD, hemimandibles were infiltrated with a series of solutions of LR White acrylic resin in ethanol (33%, 50%, 100% LR white). Hemimandibles were placed in 1 mL of each solution and left on a rotator overnight at RT before moving to the next solution. Finally, the hemimandibles were cured in LR white in gelatin capsules at 55° C overnight. Samples were cut using an Isomet (Buehler) equipped with a diamond wafering blade (Allied High Tech Product, Inc.), thinned to < 50 μm with carbon grit paper (600, 1 000 grit), and affixed to Kapton tape for μXRD.

### Optical imaging of kidneys

Optical micrographs of whole kidneys before excision of the mineral were collected with a Wild Heerbrugg M3Z microscope (Leica) equipped with a Nikon Digital Sight DS-Fi2 camera.

### Micro-computed tomography (μCT)

Whole kidneys were held in place in 2 mL centrifuge tubes with polystyrene packing peanuts and covered with 70% ethanol for μCT scanning with a Scanco (Brüttisellen, Switzerland) μCT 50 cabinet μCT scanner. Radiographs were collected at 45 kVp with a 0.1 mm Al filter. A total of 1 000 projections were collected 0° ~ 180° for a total scan time of 8 hours per sample. No frame averaging was used, and a ring artifact suppression routine with level 8 was selected in the Scanco software. Reconstructions were performed using a cone-beam convolution-backprojection in the Scanco software with an isotropic voxel size of 2 μm. Data were exported as 16-bit DICOM images, which were converted into 8-bit TIF stacks in ImageJ^58^ for further analysis.

Datasets were then analyzed using Dragonfly software. First, the data were spatially downsampled in all directions by a factor of 6 using linear interpolation (12 μm voxels). Five total datasets were registered together to reconstruct the whole kidney volume. A neural network with u-net architecture^59^ was trained in Dragonfly to differentiate calcified material, kidney tissue, and background (e.g. Eppendorf tube, air, and ethanol), using 11 hand-segmented slices spanning the length of the whole kidney. These 11 slices were divided into smaller images measuring 32 pixels × 32 pixels, and 20% was removed randomly from training to use for network validation. Training images were augmented 3 times to create a larger training pool using vertical and horizontal mirror operations and random rotations up to 180°. The network was trained using Adadelta, an extension of stochastic gradient descent^60^, with a starting learning rate of 1.0. One epoch of training encompassed passing all training images through the network in batches of 32 followed by an evaluation of network performance using the reserved validation images (validation loss). The network was trained for 50 epochs, and the learning rate was reduced by a factor of 10 if there were no improvements in the validation loss after 10 consecutive epochs. To avoid overfitting, the network was only saved at the end of an epoch if the validation loss improved.

The trained network was used to segment the kidney volume. The segmented kidney volume was corrected by hand, and pixels classified as mineral that did not border the labeled kidney volume were removed using an island filter implemented in Dragonfly. A mesh of the kidney was generated using a smoothing parameter of 4 to visualize the surface.

### Micro-Raman spectroscopy (µRaman)

Unbleached kidney mineral aggregates and a section of mature murine incisor enamel were compared to synthetic hydroxylapatite by μRaman spectroscopy using a fiber-optically coupled Raman microprobe (Horiba LabRam Confocal Raman). A 50× NIR-LWD objective was used to focus a 532 nm laser operating at 8 mW for the kidney sample and at 5.48 mW for all other samples. A transmission grating (1 800 gratings per mm) split the collected signal before delivering it to a CCD detector (HORIBA Jobin-Yvon, Kyoto, Japan). Spectra were collected from 400 to 3 000 cm^−1^ with a spectral resolution of 1 cm^−1^. Spectral acquisition time was 2× 15 s–3× 15 s per spectral window, for a total of approximately 4.5 minutes for each location sampled. Inspection of the sample surface did not reveal indications for radiation damage. Data were recorded as LabSpec 6 spectra and video files and converted to CSV and TIF files using LabSpec 6 software. Using Matlab, quadratic backgrounds were subtracted from all spectra using a background estimation code^61^ with a threshold of 0.05 and an asymmetric quadratic cost function. For the kidney, background was subtracted from each spectral window separately. Data were then normalized to the maximum intensity of the spectrum, and a moving average with a sliding window of 10 points was calculated. Peaks were identified using peak picking as implemented in the Matlab function findpeaks^@^, using a minimum prominence parameter of 100. For a comparison with geological samples of calcium phosphate minerals, spectra from the RRUFF online database^62^ were used (fluorapatite, R040098; collinsite, R060140; hydroxylapatite, R050512; monetite, R070259; isokite, R070526; brushite, R070554; whitlockite, R070675).

### Scanning electron microscopy with energy dispersive spectroscopy (SEM-EDS)

SEM-EDS was performed using a JEOL JSM-7900F Schottky Field Emission microscope (JEOL, Ltd., Tokyo, Japan). Uncoated samples were affixed to aluminum SEM stubs using carbon tape. Samples were analyzed in low vacuum mode at 30 Pa (N_2_) with an accelerating voltage of 15 kV, emission current of ~60 μA, and working distance of 10 mm. An Oxford Ultimax 65 EDS detector (Oxford Instruments, Abingdon, UK) was used with Aztec software (Oxford Instruments) to collect EDS point spectra using 1 024 or 2 048 channels in a 20 keV detection range with pulse pile-up correction enabled. Data collection proceeded until at least 500 000 counts had been recorded at each position on the sample. Data were exported as text files using Aztec software and plotted in Matlab. Emission lines for elements of interest were extracted from Thompson and Vaughan, 2001^63^.

### Synchrotron X-ray microdiffraction (µXRD)

µXRD was performed at beamline 34ID-E of the Advanced Photon Source (APS) at Argonne National Laboratory (Lemont, IL). Monochromatic X-rays (17.0 keV or 20.0 keV) were focused with Kirkpatrick–Baez mirrors down to a beam size of 250 × 280 nm^2^ (HxV), and 2D diffraction patterns were collected in transmission geometry using MAR165 area detector. The sample was attached to a stainless-steel holder with Kapton tape and mounted on a XYZ-translational stage at the beamline. The sample holder was positioned in the focal plane of the X-ray beam such that the normal to the sample surface was rotated about the positional X-axis (storage ring inboard-outboard axis) -45° relative to the incident beam (Z-direction). Twenty five diffraction patterns from one mineral deposit in one kidney were recorded, sampling on a square grid with a pitch of 5 µm in the X- and Y-direction, integrating over 10 s or 20 s per pattern. Reference patterns were collected from a wild-type mature murine incisor and surrounding jawbone in 1 μm steps (25 patterns each for enamel and dentin, 21 patterns for bone). Calibration patterns were collected for powdered ceria and synthetic hydroxyapatite.

An Initial geometry (beam center coordinates, sample-to-detector distance, detector tilt and rotation) was refined for the ceria standard using Fit2D^64^. Additional refinements to the beam center on this pattern were performed using custom code in Matlab^47,65^. Radial 1D profiles for kidney, bone, dentin, and enamel were calculated in Fit2D using the refined geometry parameters and exported as chiplot files. For full 1D radial plots, a quadratic background was subtracted using background correction code in Matlab with a sensitivity of 0.01 and an asymmetric truncated quadratic cost function. *d*-spacings for the 002, 121, and 030 peaks were extracted for hydroxylapatite, PDF card 00-064-0738^66^ and overlaid with the data.

For individual peak fitting for Scherrer analysis, the LIPRAS graphical user interface in Matlab was used to obtain a linear background estimate around the 002, 121, 112, 030, and 022 reflections. These peaks were fitted, after subtraction of the linear background, using Pseudo-Voigt models^67^. The 002 peak was fit separately from the 121, 112, 030, and 022 reflections, which were fit simultaneously. The position (*θ*_*hkl*_) and full width at half-maximum intensity (FWHM, *B*_*hkl*_) were extracted.

Instrumental contributions to peak broadening due to focused beam divergence were determined using a Si single crystal. The resulting 2D diffraction pattern was converted to a 1D radial profile in Fit2D and fit using custom Matlab code^47,65^. The FWHM of the Si standard (*B*_*I*_ = 0.066˚) was taken as the instrumental broadening and subtracted to determine the corrected broadening:

1 $${B}_{{\rm{corr}}}=\sqrt{{{B}_{{\rm{hkl}}}}^{2}-{{B}_{{\rm{I}}}}^{2}}$$

Contributions to peak broadening due to microstrain were neglected. The crystallite size was then calculated from the corrected broadening of the 002, 121, and 030 reflection peaks using the Scherrer equation^45,46^.

2 $${D}_{{hkl}}=K\frac{\lambda }{{B}_{{\rm{corr}}}\cos {\theta }_{{\rm{hkl}}}}$$

where *D*_*hkl*_ is the coherent domain length perpendicular to the hkl planes (e.g. *D*_002_), *K* is the crystallite shape factor (*K* = 1), and λ is the X-ray wavelength used (λ = 0.729 3 Å).

Statistical tests were performed in Python, using kruskal() from the scipy package (v 1.2.1)^68^, and posthoc_dunn() from the scikit_posthocs package (v0.6.7)^69^.

### STEM-in-SEM Imaging

Bright-field STEM-in-SEM imaging was carried out using a JEOL JSM-7900F Schottky Field Emission SEM equipped with a STEM-converter sample holder, using an accelerating voltage of 20 kV, probe size 8, emission current of 61.4 pA, and a working distance of 4.88 mm. Secondary electron signal reflected from the holder’s gold mirror were collected using the lower electron detector.

### Supplementary information

Supplemental material

## Data Availability

The data that support the findings of this study are available from the corresponding author upon reasonable request. The accession number for the bulk RNA-Seq data reported in this paper is GEO: GSE220686.
