# Supplementary material for: Adult dental epithelial stem cell-derived organoids deposit hydroxylapatite biomineral
Source: Int J Oral Sci. 2023 Dec 7;15:55. doi: 10.1038/s41368-023-00257-w (PMC10703793; doi:10.1038/s41368-023-00257-w)
Supplement: Supplementary file 1 — Supplemental material [file 41368_2023_257_MOESM1_ESM.docx]

**Adult Dental Epithelial Stem Cell-Derived Organoids Deposit Hydroxylapatite Biomineral**

Hyun-Yi Kim, Victoria Cooley, Eun-Jung Kim, Shujin Li, Jong-Min Lee, Dina Sheyfer, Wenjun Liu, Ophir D. Klein, Derk Joester and Han-Sung Jung

**Supplementary Figure and Figure Legends**

**
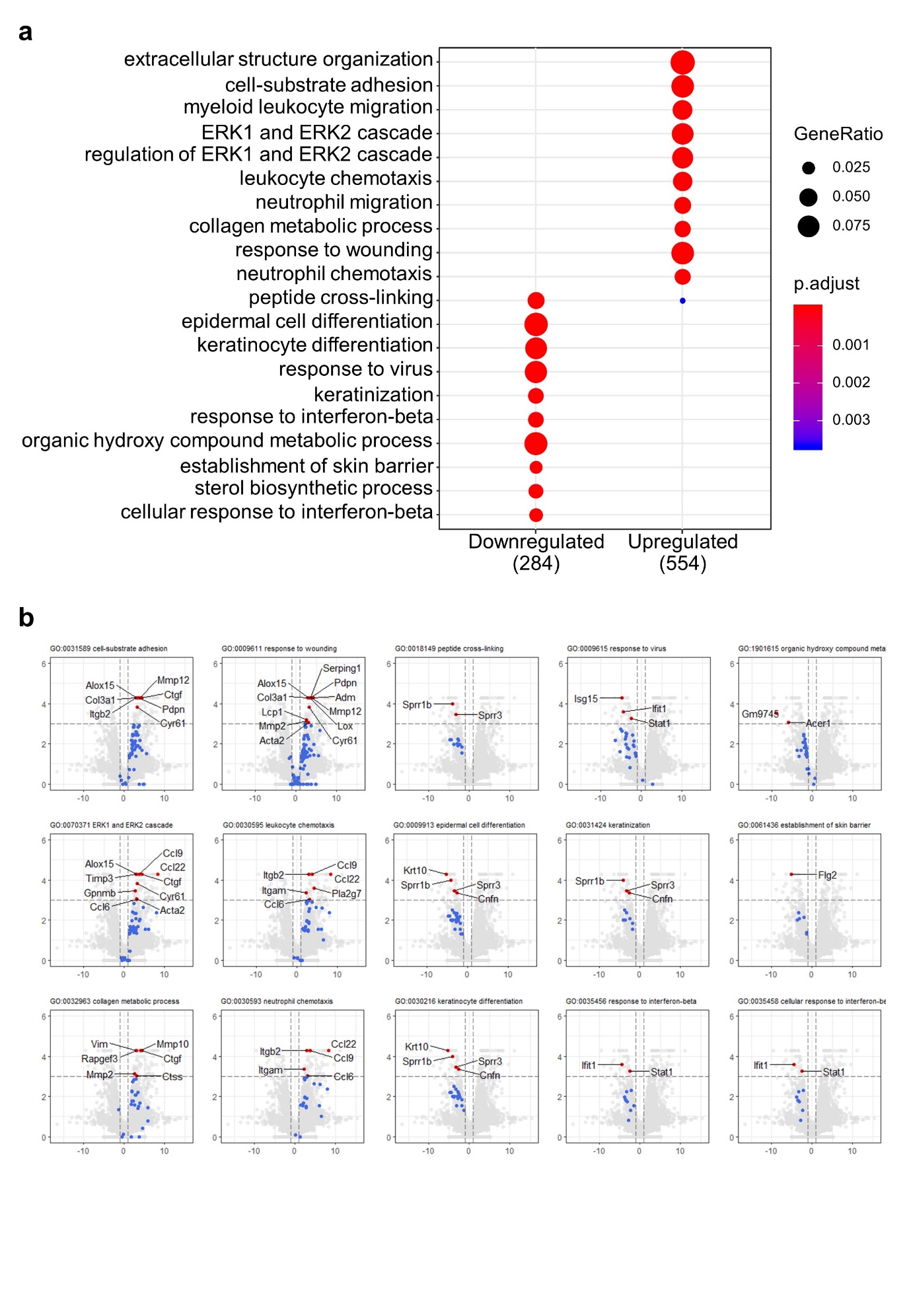
**

**Fig. S1** Comparison of DEOs grown in +NJ and +NFJ, Related to Fig. 1. **a** A dot plot of GO over-representation results of downregulated and upregulated DEGs in DEOs grown in +NFJ compared with +NJ. The color and size of dots indicate the p-value and gene ratio of each representative GO term. **b** Volcano plots of differentially expressed genes (DEGs) of DEOs grown in +NFJ compared with +NJ. The indicated GO term-related genes are displayed as red (|fold change| > 2 and p-value < 0.001), blue (|fold change| < 2 or p-value > 0.001), and gray (not related to the indicated GO term) dots. Vertical dashed lines indicate -2- and 2-fold changes. A horizonal dashed line indicates a p-value of 0.001.


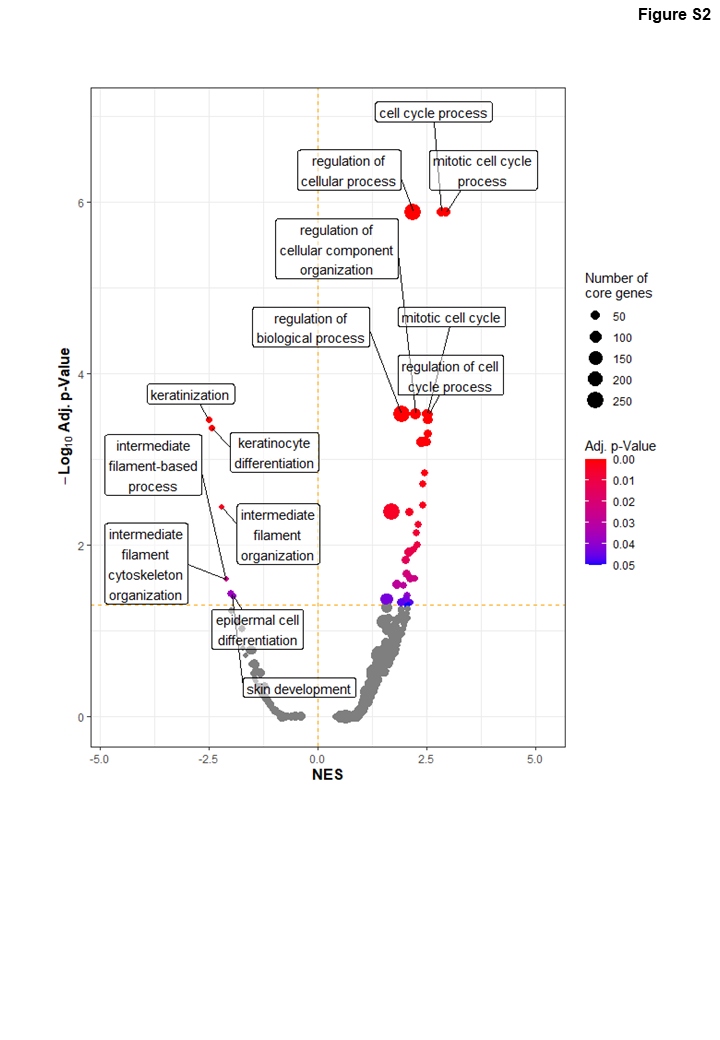
**Fig. S2** Gene set enrichment analysis (GSEA) for significantly regulated genes oof organoids grown +NF compared to +N. The result of GSEA visualized using bubble plots. Size of dots indicates number of core genes for enriched GO terms. The color of dots shows Benjamini-Hochberg adjusted p-Value of enriched GO terms. Vertical dashed lines indicate 0 of normalized enrichment score (NES). A horizonal dashed line indicates a p-value of 0.05.


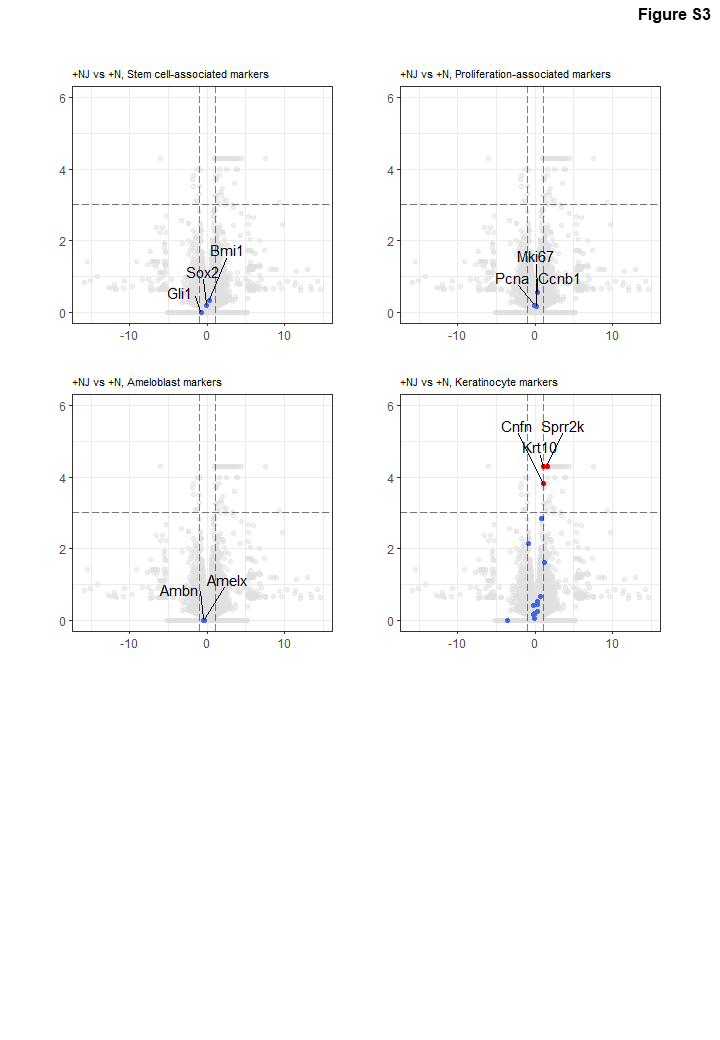


**Fig. S3** Volcano plots of differentially expressed genes (DEGs) of DEOs grown in +NJ compared with +N. The indicated biological function-related genes are displayed as red (|fold change| > 2 and p-value < 0.001), blue (|fold change| < 2 or p-value > 0.001), and gray (not related to the indicated GO term) dots. Vertical dashed lines indicate -2- and 2-fold changes. A horizonal dashed line indicates a p-value of 0.001.

**
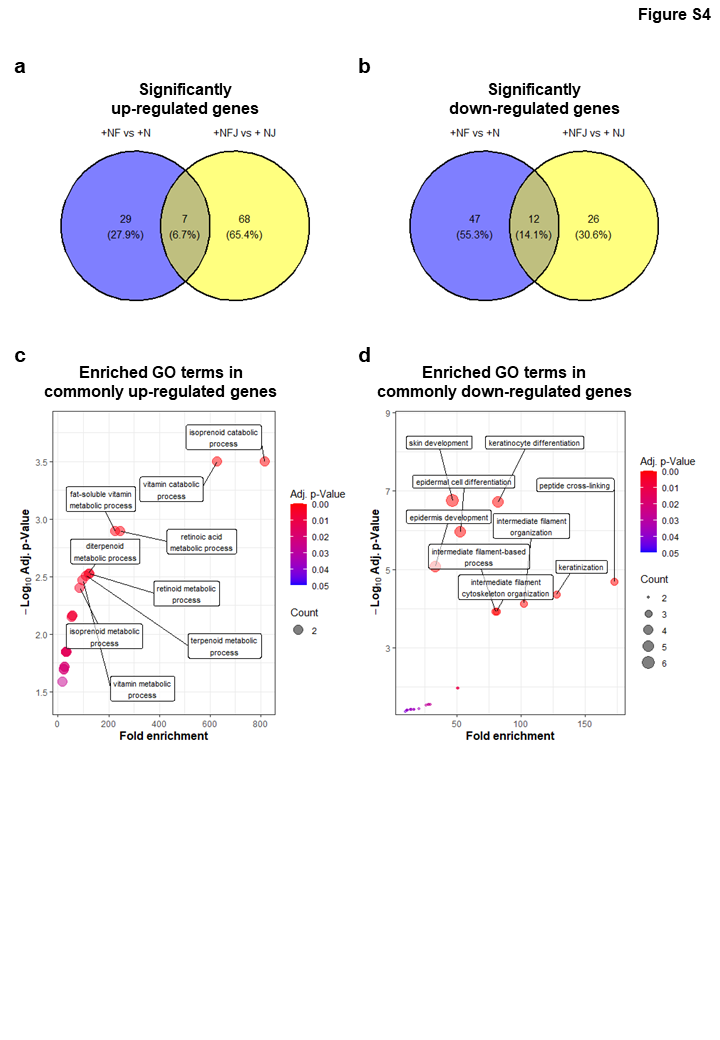
**

**Fig. S4** Comparison of significantly up- and down-regulated genes in DEOs grown in +NF and +NFJ compared to +N. **a-b** Venn diagrams showing the number of commonly up- or down-regulated genes. **c-d** Bubble plots of the gene ontology analysis result for commonly up-regulated genes. Size of dots indicates number of genes for enriched GO terms. The color of dots shows Benjamini-Hochberg adjusted p-Value of enriched GO terms.

**
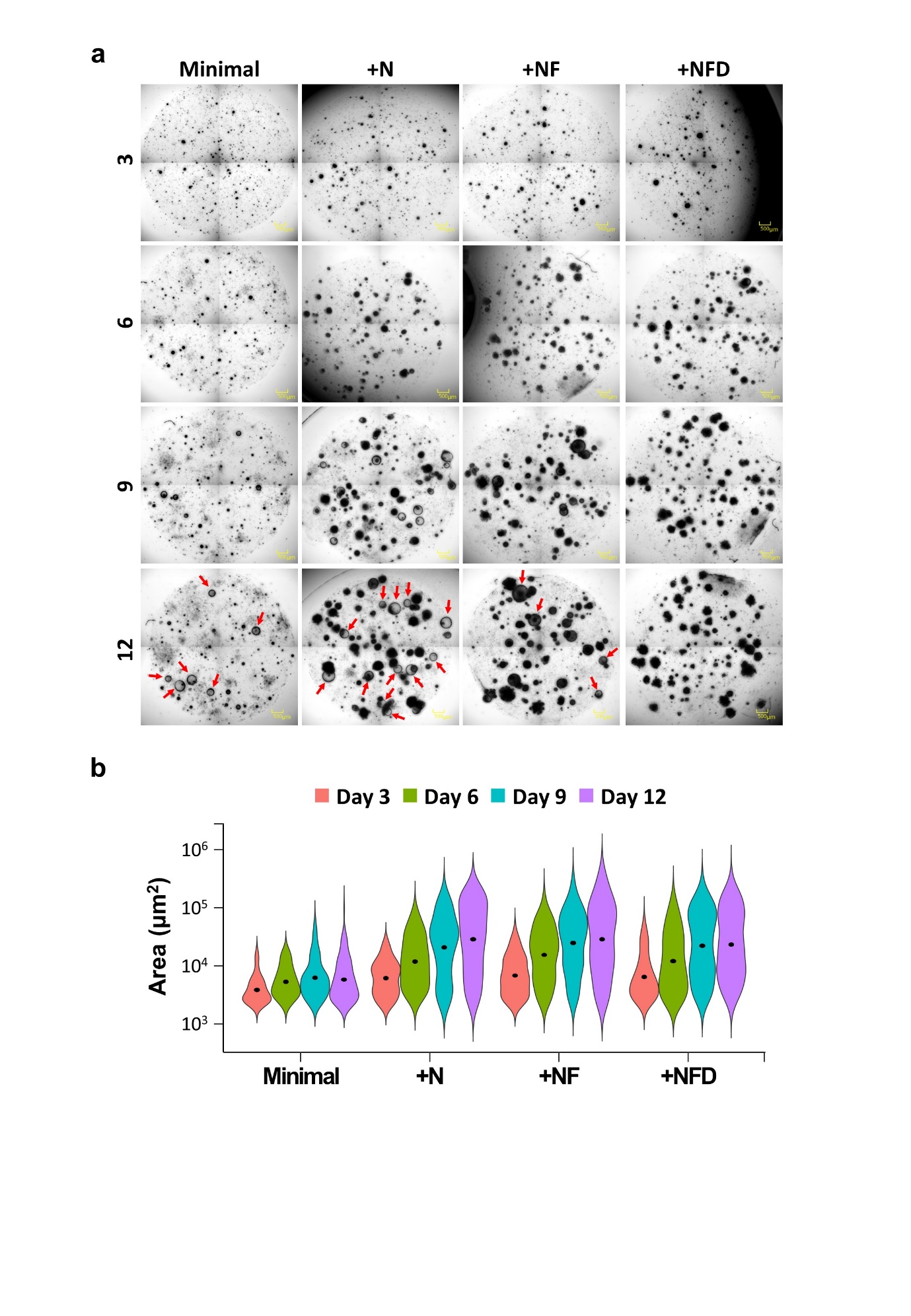
**

**Fig. S5** Organoid growth as a function of media composition. Adult dental epithelial stem cells (aDESCs) isolated were embedded into Matrigel and grown in for 3, 6, 9, and 12 days in the in the media indicated (minimal, +N, +NF, and +NFD). **a** Maximum intensity projection of a z-stack of DIC images. Red arrows indicate hollow spheres. **b** Size distributions of organoids at day 3, 6, 9, and 12 for culture in minimal, +N, +NF, and +NFD media shown as a violin plot. The mean of each distribution is indicated (black circle).


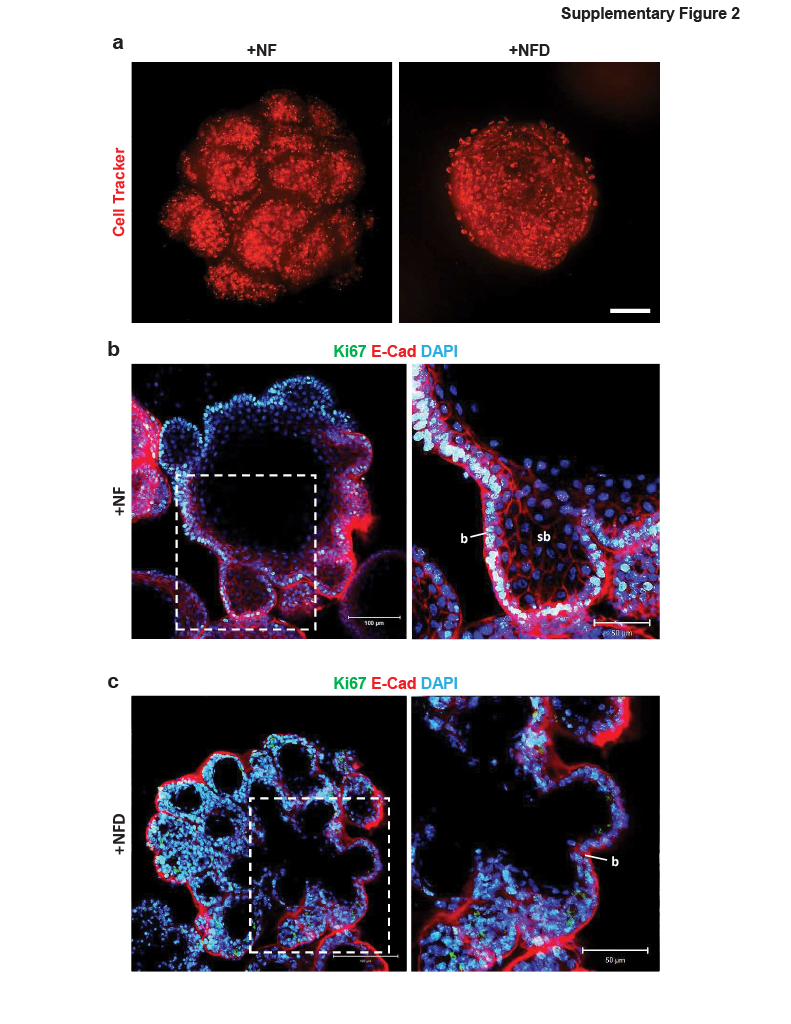


**Fig. S6** Effect of Notch signaling inhibitor dibenzazepine on organoids. **a** Maximum intensity projection of z-stacks of confocal images of whole organoid grown in +NF and +NFD media stained with a live cell fluorescent dye, Cell Tracker Red CMTPX. Scale bar = 50 μm. **b-c** Maximum intensity projection of z-stack of confocal images of a whole organoids grown in +NF (**b**) and +NFD (**c**) media immuno-stained for Ki67 (red) and E-cadherin (E-cad, green), and counter-stained with DAPI (blue). The image on the right is a closeup of the region indicated by the dashed box in the image on the left.

**
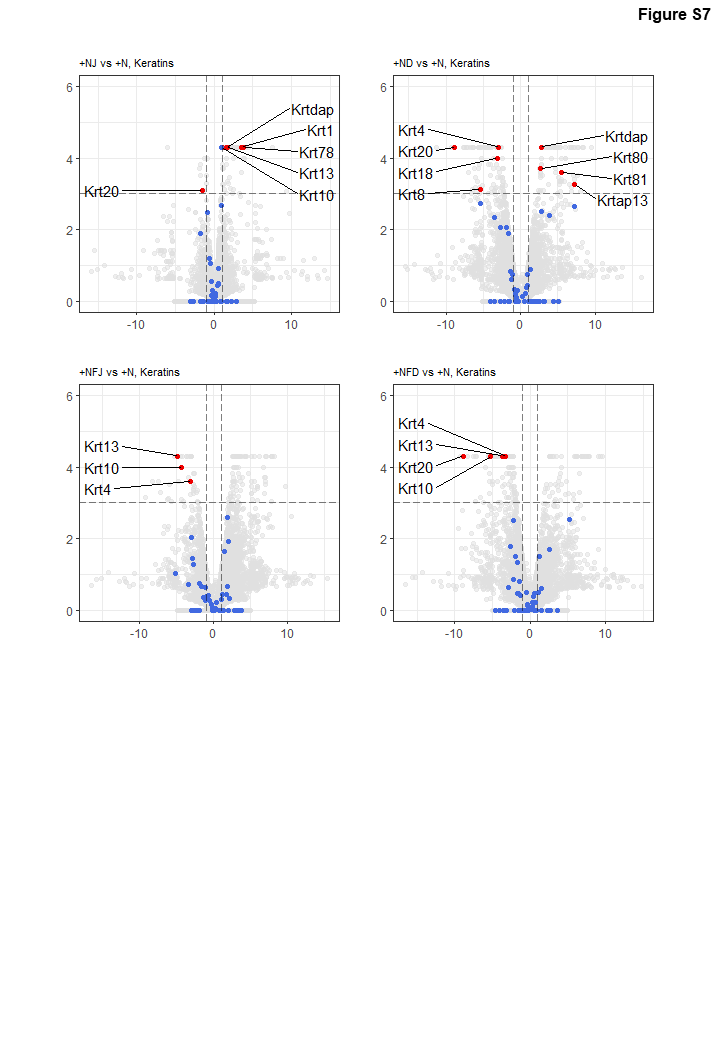
**

**Fig. S7** Volcano plots of DEGs of DEOs grown in +NJ, +ND, +NFJ, and +NFD compared with +N. The indicated Krt-related genes are displayed as red (|fold change| > 2 and p-value < 0.001), blue (|fold change| < 2 or p-value > 0.001), and gray (not related to the indicated GO term) dots. Vertical dashed lines indicate -2- and 2-fold changes. A horizonal dashed line indicates a p-value of 0.001.**
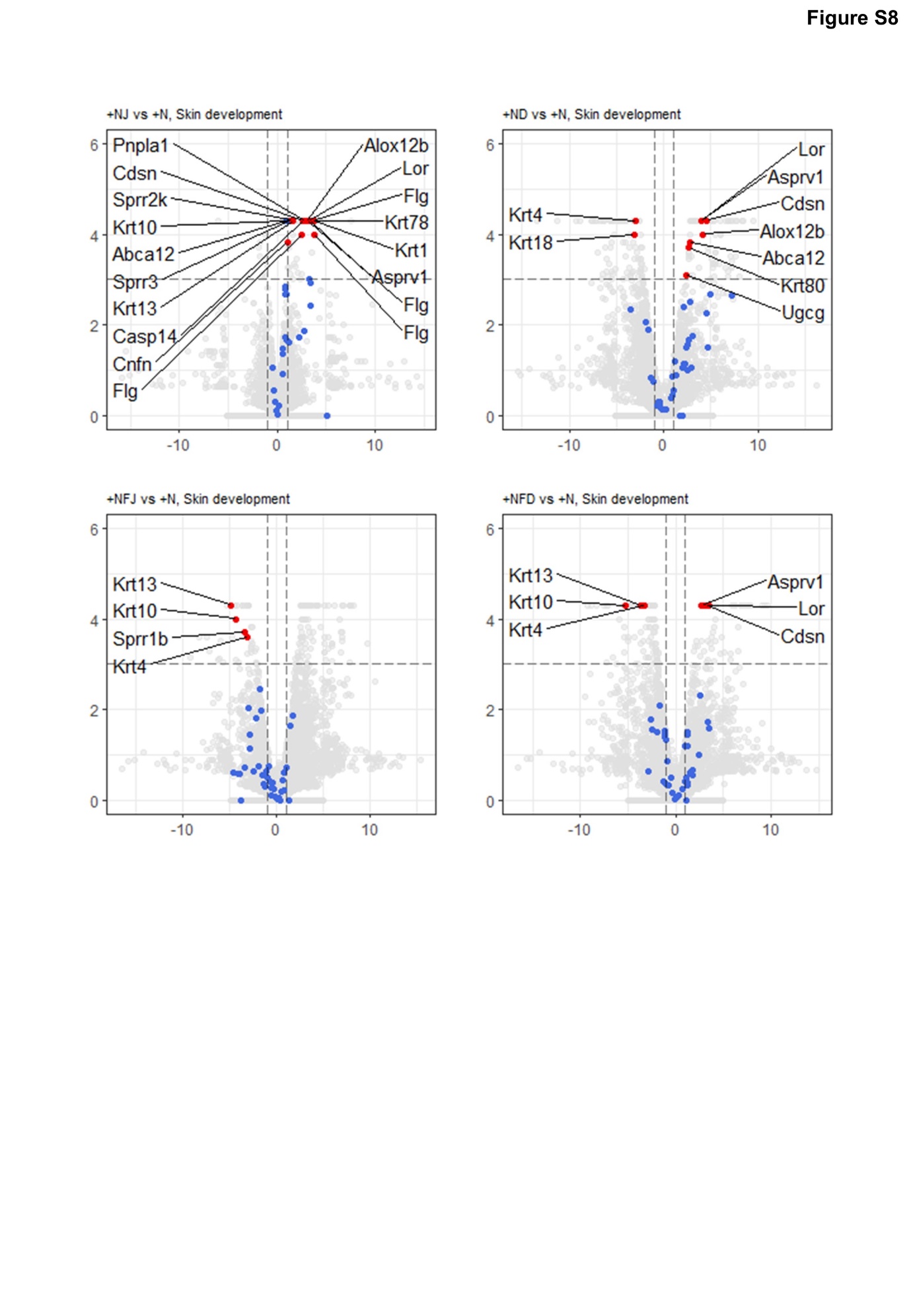
**

**Fig. S8** Volcano plots of DEGs of DEOs grown in +NJ, +ND, +NFJ, and +NFD compared with +N. The indicated skin development-related genes are displayed as red (|fold change| > 2 and p-value < 0.001), blue (|fold change| < 2 or p-value > 0.001), and gray (not related to the indicated GO term) dots. Vertical dashed lines indicate -2- and 2-fold changes. A horizonal dashed line indicates a p-value of 0.001.

**
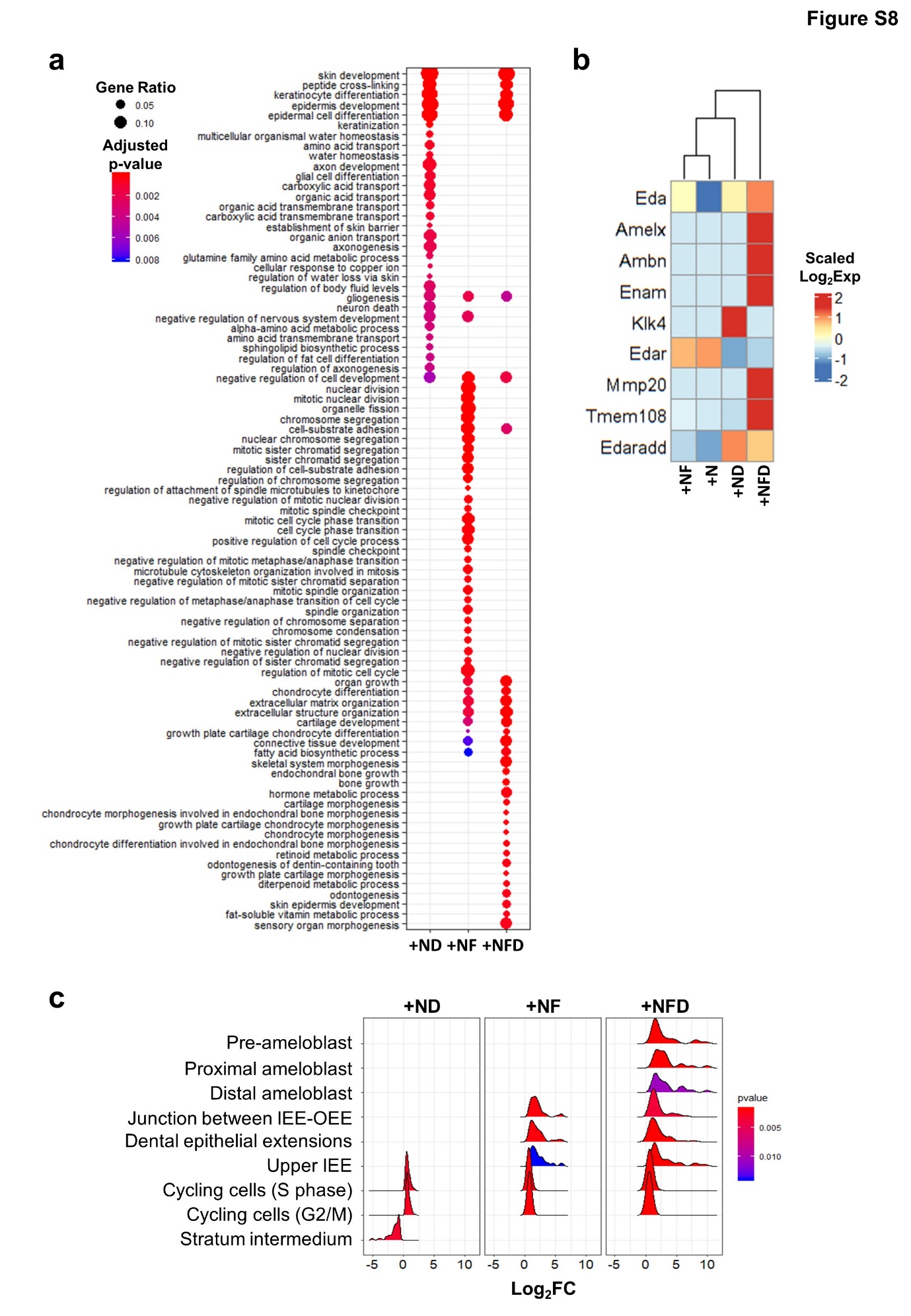
**

**Fig. S9** Transcriptomic analysis of organoids grown in various medium conditions. **a** A dot plot visualizing gene ontology (GO) overrepresentation results for upregulated genes in organoids grown in +ND, +NF, and +NFD media compared with organoids grown in minimal media. Gene ratio means number of upregulated genes found in a gene set over total number of upregulated genes. **b** A heatmap visualizing transformed and scaled expression (Scaled Log_2_Exp) of ameloblastic markers in organoids grown in +NF, +N, +ND, and +NFD media. **c** Ridgeline plot of a gene set enrichment analysis of upregulated genes using gene sets described in Sharir et al., 2019. The height of plot indicates density of probability that a gene of a gene set has specific fold change (Log2FC).


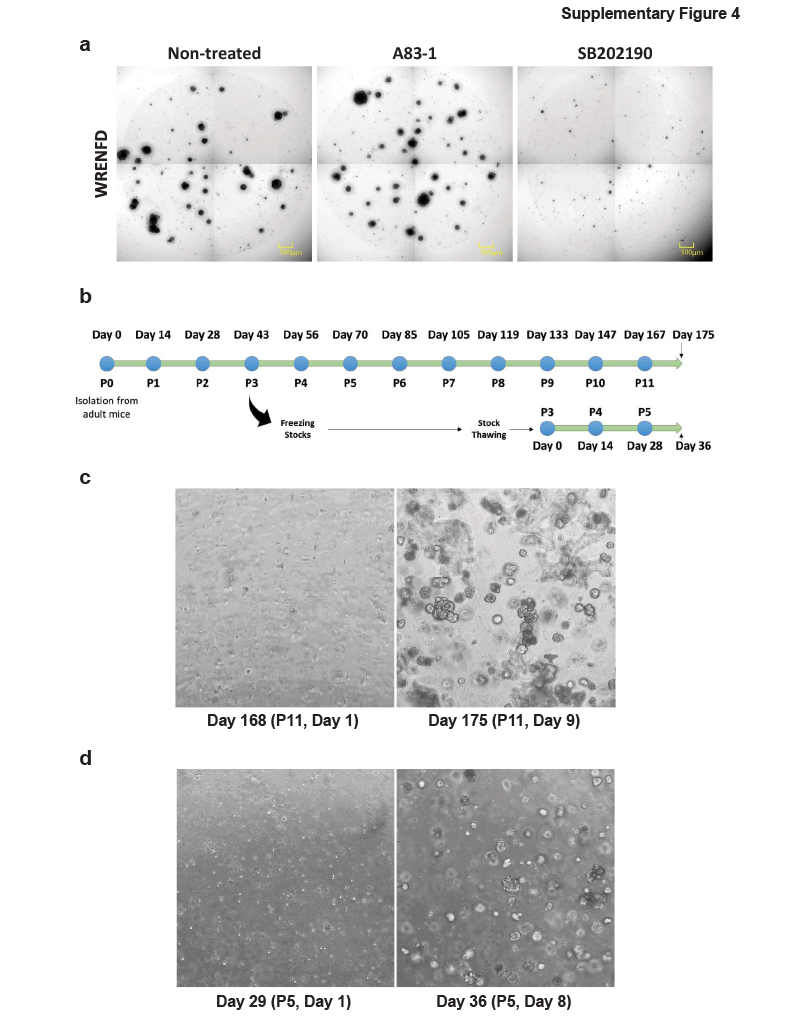


**Fig. S10** Long-term culture, freezing and thawing of DEOs**. a** Maximum intensity projection of z-stack of DIC images of DEOs grown in +NFD supplemented with A83-1 or SB202190 for 12 days. **b** Schematic depicting the long-term culture and freezing, storing and thawing of DEOs. **c** Bright field images of long-term-cultured DEOs. **d** Bright field images of growing DEOs after freezing, storing, and thawing.


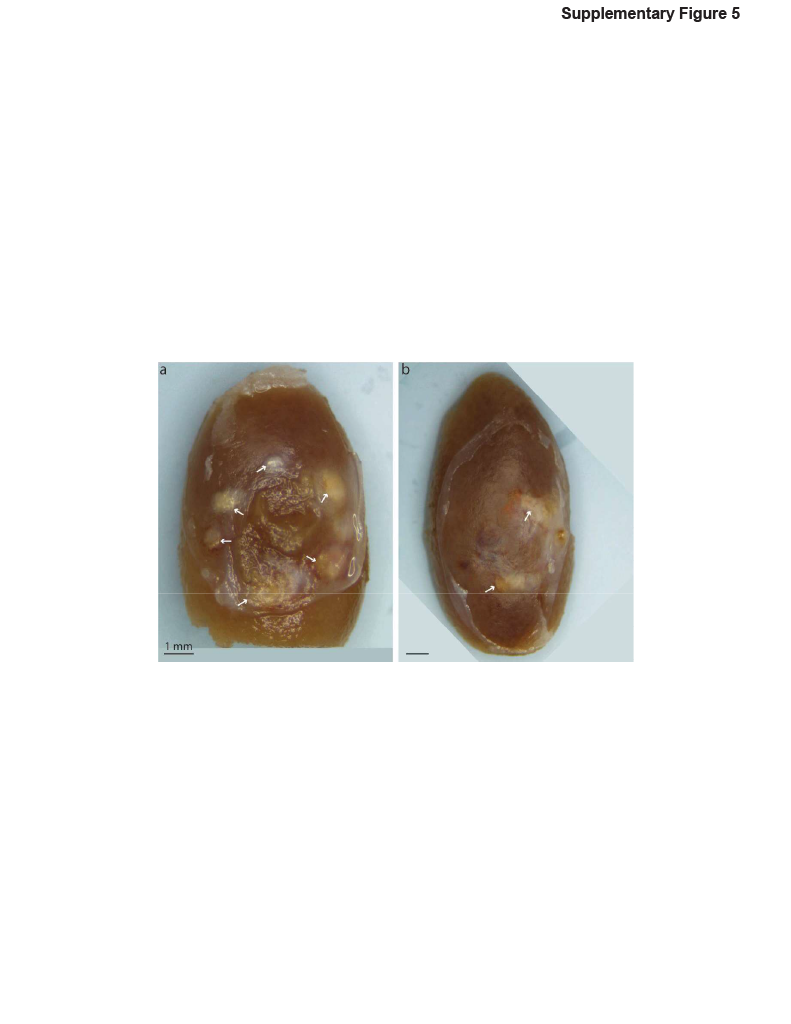


**Fig. S11** Optical microscope images of mouse kidneys harvested 8 weeks after subcapsular implantation of DEOs. Note whitish masses (white arrows). Scale bars = 1 mm.


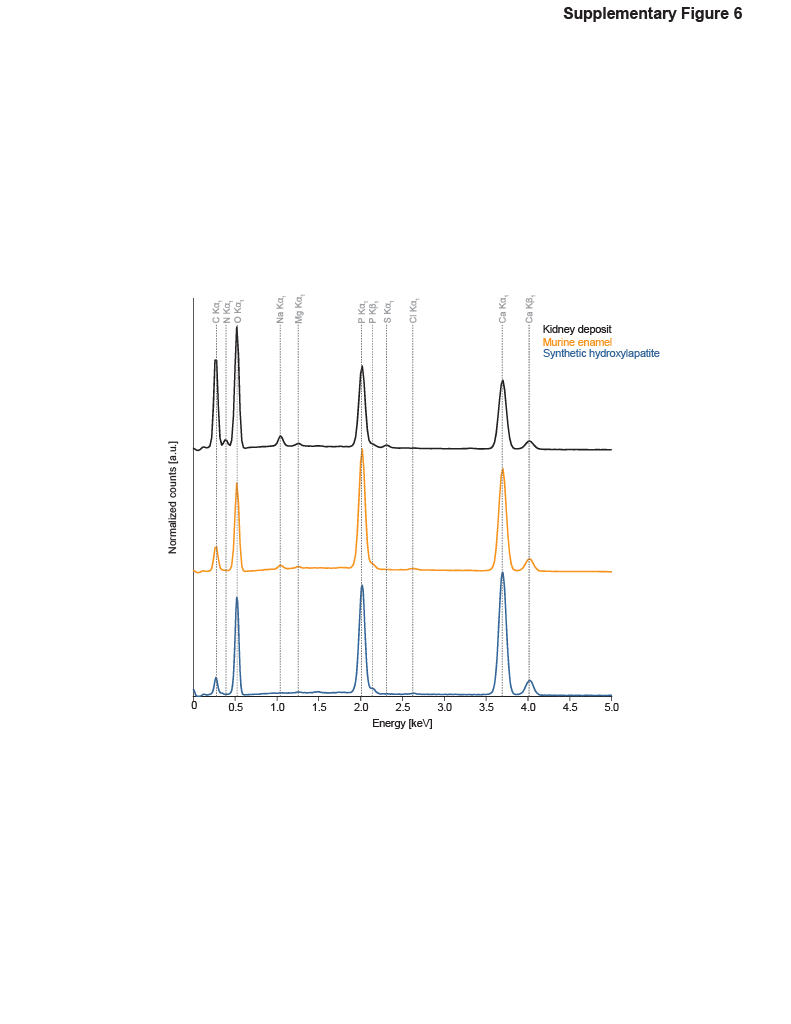


**Fig. S12** Elemental analysis by energy-dispersive X-ray spectroscopy (EDS). Representative EDS spectra for mineral deposits in the kidney (black), murine enamel (yellow), and synthetic hydroxyapatite (blue). Photon energies of principal K-shell emission lines for identified elements are indicated.

**
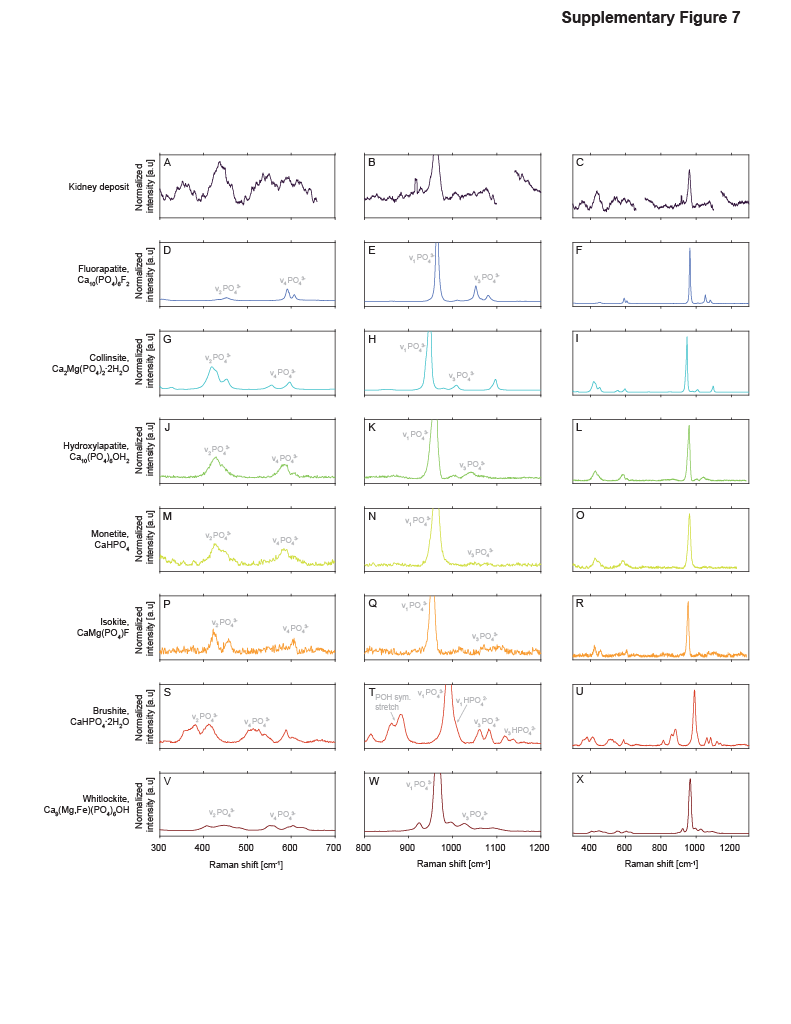
**

**Fig. S13** Comparison of geological calcium phosphate Raman spectra. Spectra are provided for the kidney deposit (**a**-**c**) and geological samples of fluoprapatite (**d**-**f**), collinsite (**g**-**i**), hydroxylapatite (**j**-**l**), monetite (**m**-**o**), isokite (**p**-**r**), brushite (**s**-**u**), and whitlockite (**v**-**x**). Close-up views are provided for the PO_4_^3-^ bending region, 300-700 cm^-1^ (**a**, **d**, **g**, **j**, **m**, **p**, **s**, **v**), and the PO_4_^3-^ stretching region, 800-1200 cm^-1^ (**b**, **e**, **h**, **k**, **n**, **q**, **t**, **w**). Vibrational modes identified in the literature are labeled for each mineral^1-7^. Full spectra are also provided (**c**, **f**, **i**, **l**, **o**, **r**, **u**, **x**).

**
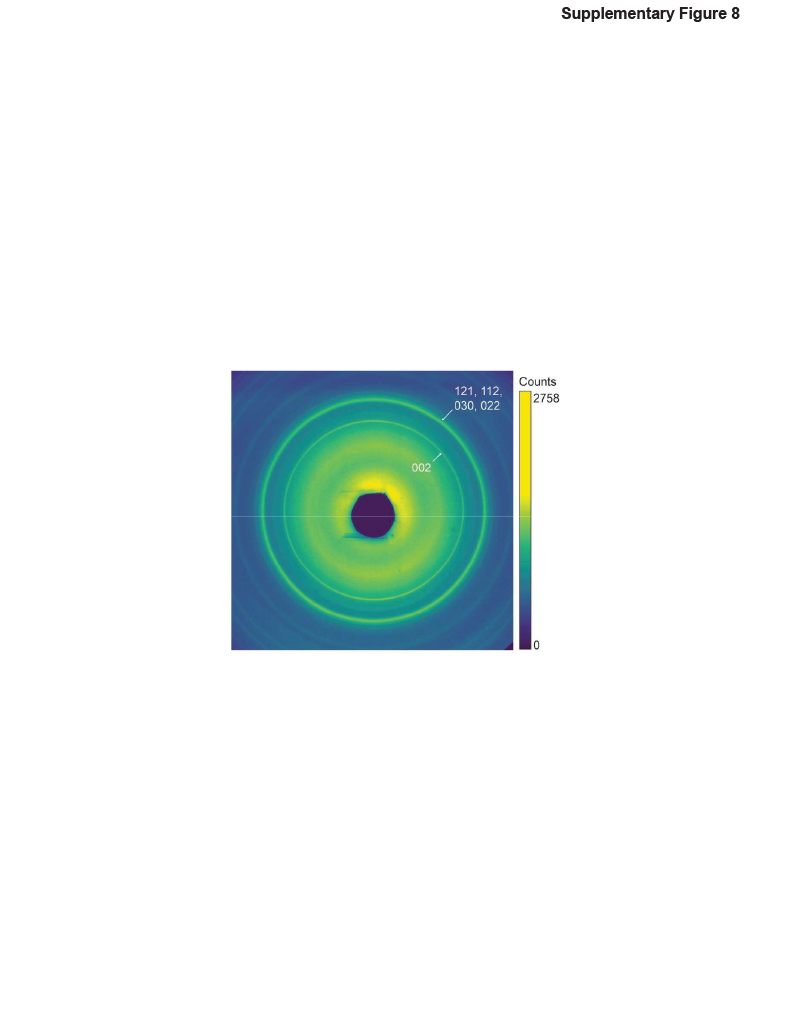
**

**Fig. S14** Representative 2D diffraction pattern for mineral deposits in the kidney. Full concentric rings indicate that no crystallographic texture (preferential orientation) is present in the sample. Reflections of interest (002 and the quadruplet 121, 112, 030, 022) are labeled.


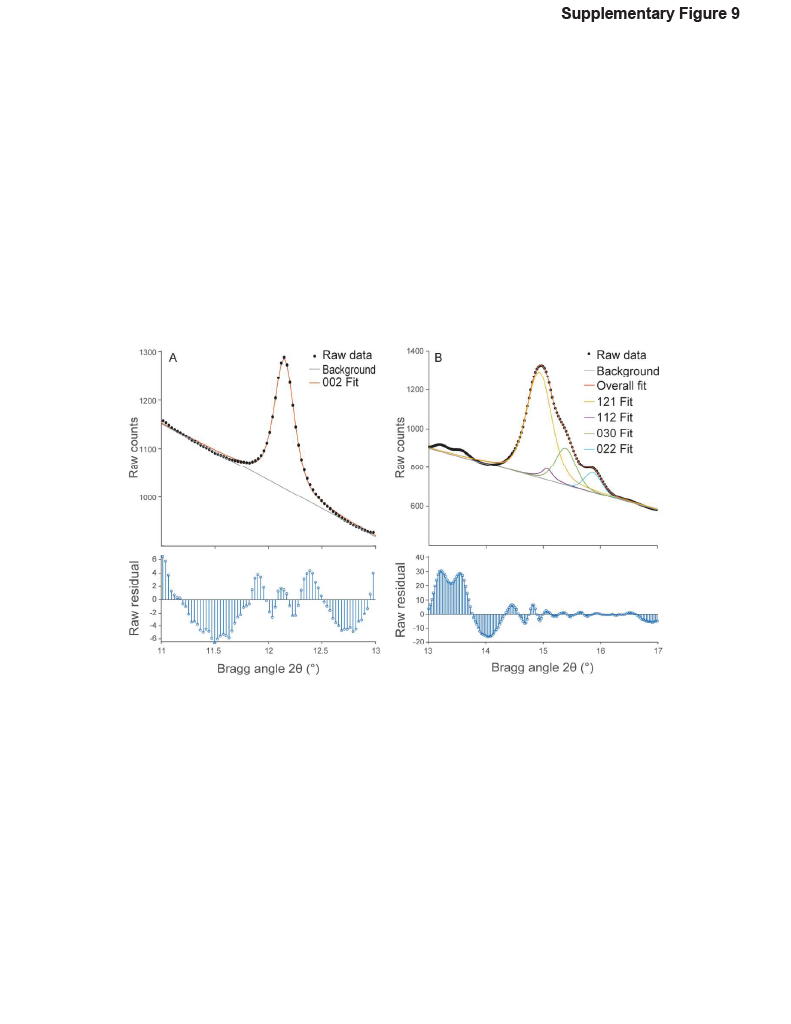


**Fig. S15** Fit results for a typical X-ray diffraction pattern collected from mineral deposits in the kidney. **a** The diffracted intensity in the region $2\theta=11-13˚$ was fitted with a linear function for the background and a Pseudo-Voigt model for the 002 reflection. **b** The diffracted intensity in the region $2\theta=13-17˚$ was fitted with a linear background and four Pseudo-Voigt profiles simultaneously for the quadruplet (121, 112, 030, and 022). Residuals for both fits (blue stem plot) are shown on separate axes.


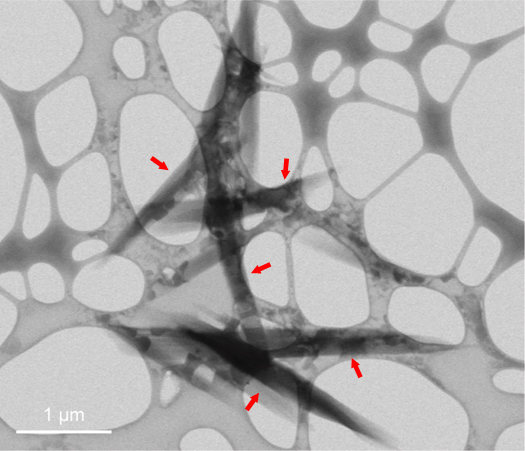


**Fig. S16** STEM-in-SEM image using brightfield contrast showing crystallites with high aspect ratio (red arrow).


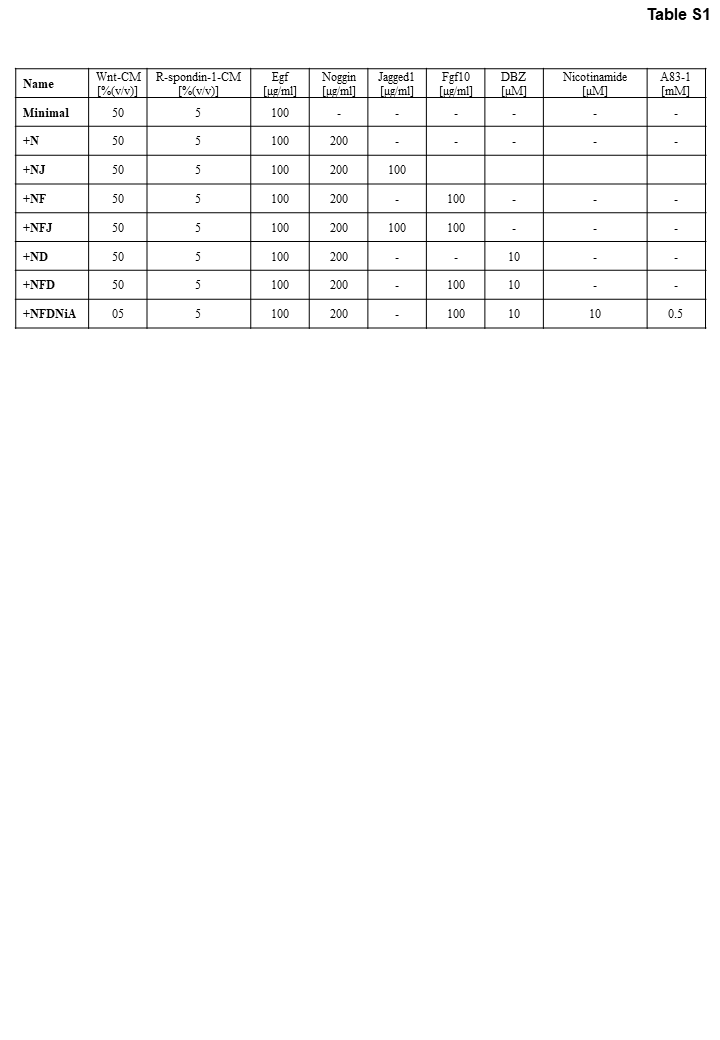


**Table S1** Composition of media. CM, conditioned medium


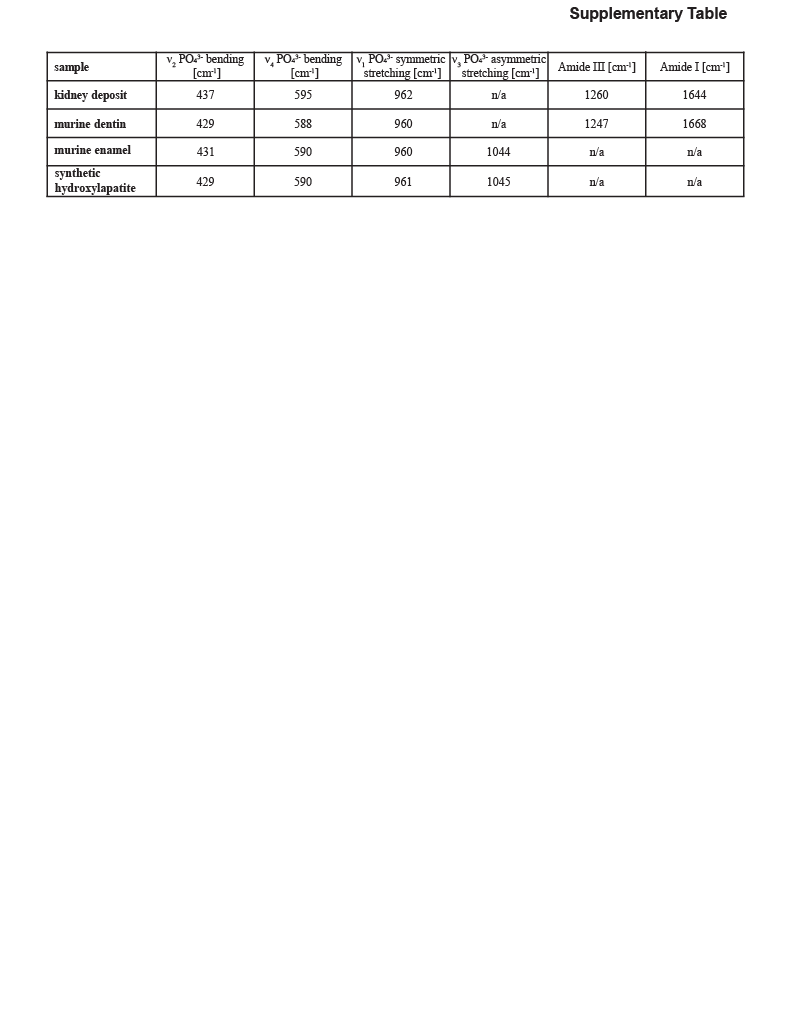


**Table S2** Characteristic features in μRaman spectra. Assignment based on De Aza et al., 1997^8^ and Rygula et al. 2013^9^.


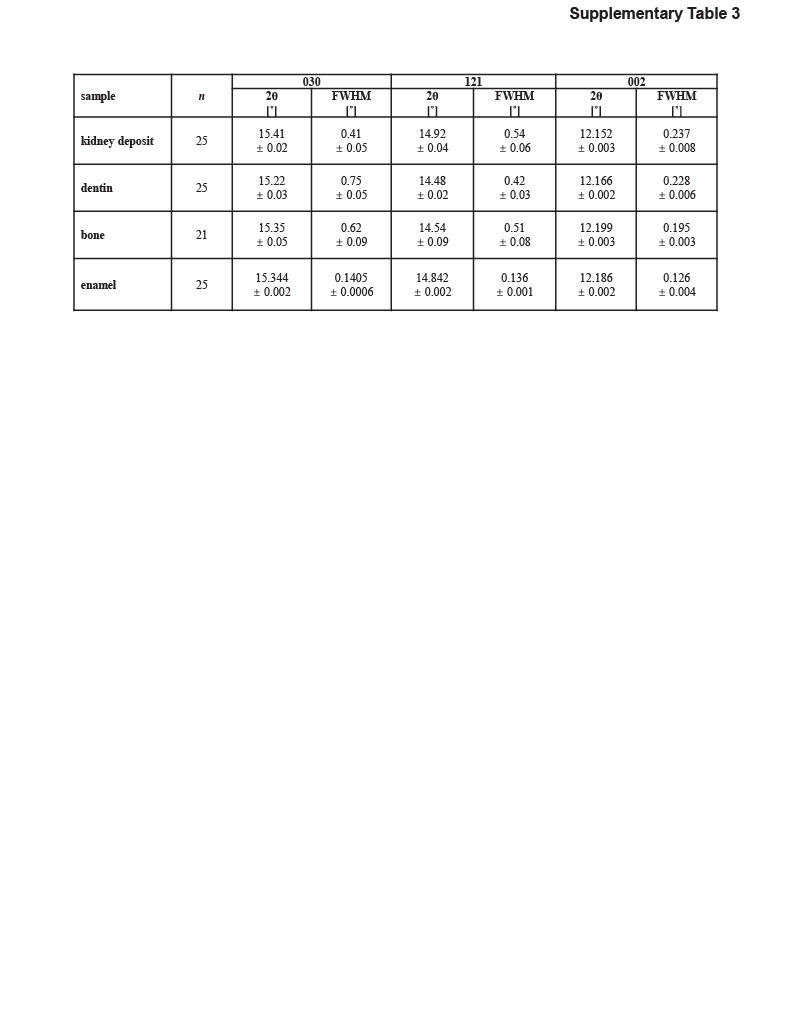


**Table S3** Characteristic features in µXRD patterns. Mean values and standard deviation for fitted Bragg angles (2θ) and full width at half maxima (FWHM) for three OHAp reflections (030, 121, and 002). *n* is the number of technical replicates. There was one biological replicate only.


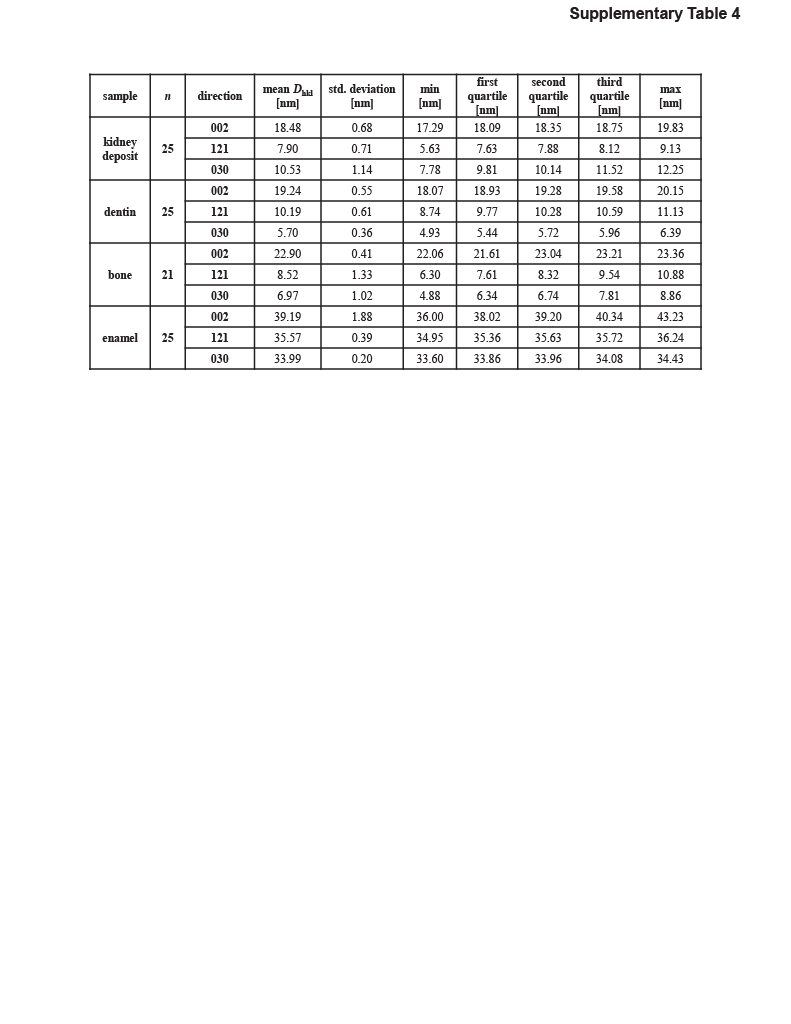


**Table S4** Distribution of crystallite size. Statistical data describing the distribution of crystallite sizes determined by Scherrer analysis of µXRD data (see also Table S3 and Fig. 6k). *n* is the number of technical replicates. There was one biological replicate only.


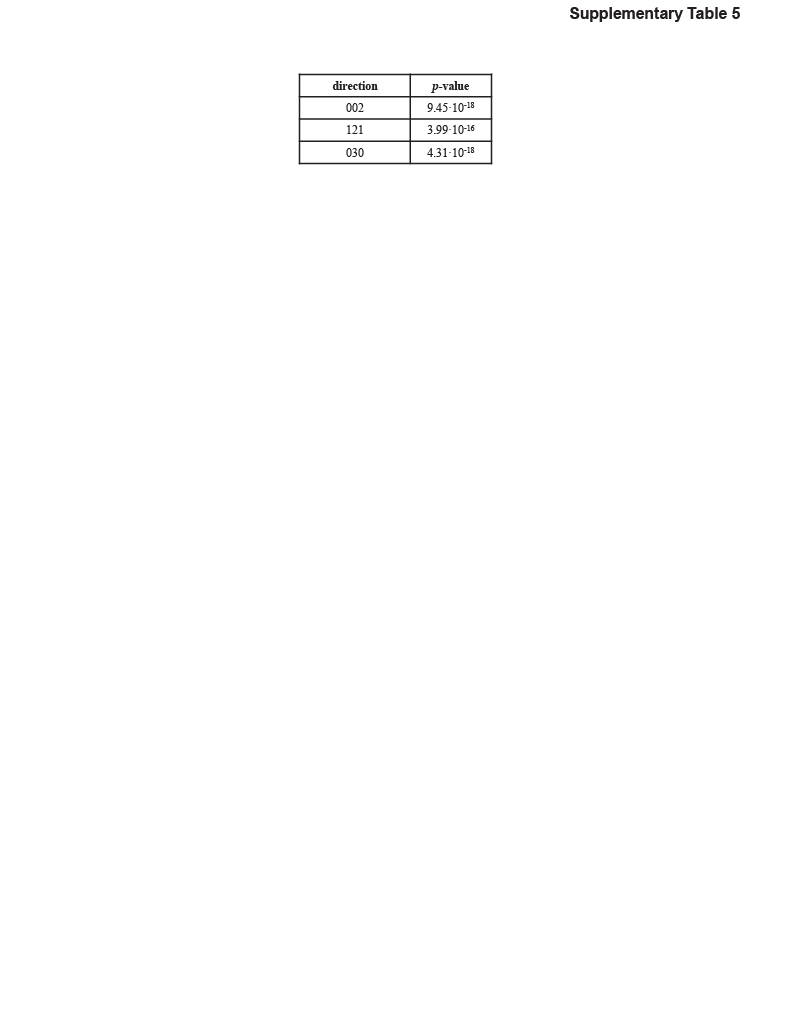


**Table S5** Statistical significance of difference of mean crystallite sizes. For each direction, a Kruskal-Wallis test rejects the null hypothesis (p < 0.05) that the *D_hkl_* values for OHAp deposits in the kidney, and OHAp in bone, dentin, and enamel are all drawn from the same distribution. For pair-wise follow-up test, see Table S6.


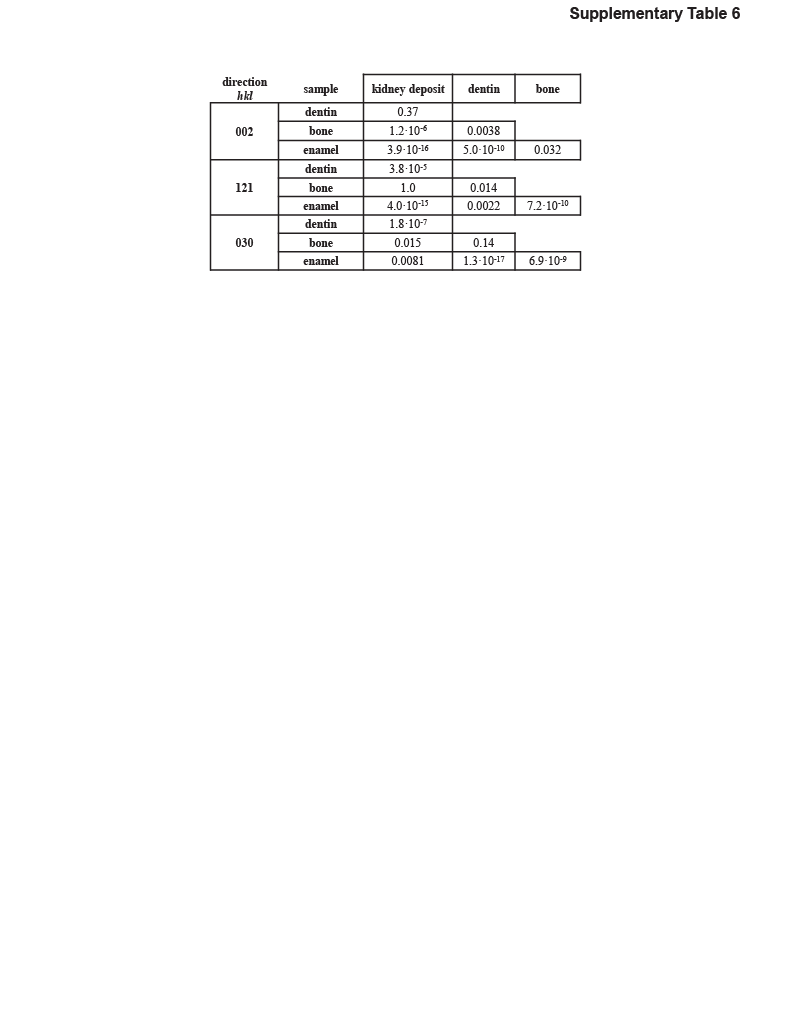


**Table S6** Significance of multiple comparison of crystallite sizes. Pairwise comparisons of samples using Dunn’s test with Bonferroni correction for multiple comparison. For mean *D_hkl_* values see Table S4, for box plot see Fig. 6k.

**Supplementary References**

1. Walters, M., Leung, Y., Blumenthal, N., Konsker, K. & LeGeros, R. A Raman and infrared spectroscopic investigation of biological hydroxyapatite. *Journal of inorganic biochemistry* **39**, 193-200 (1990).

2. Tvrdy, J., Sejkora, J., Rosseel, P. & Dolnicek, Z. Ferraioloite from the Sitio do Castelo mine, Folgosinho (Guarda, Portugal), description and Raman spectroscopy. *Journal of Geosciences* **66**, 139-146 (2021).

3. Leroy, G. et al. Polarized micro-Raman study of fluorapatite single crystals. *Applied Spectroscopy* **54**, 1521-1527 (2000).

4. Frost, R.L., Xi, Y., Scholz, R., Belotti, F.M. & Lopez, A. Infrared and Raman spectroscopic characterization of the phosphate mineral fairfieldite–Ca2 (Mn2+, Fe2+) 2 (PO4) 2· 2 (H2O). *Spectrochimica Acta Part A: Molecular and Biomolecular Spectroscopy* **106**, 216-223 (2013).

5. Frost, R.L. et al. Raman spectroscopy of synthetic CaHPO4· 2H2O–and in comparison with the cave mineral brushite. *Journal of Raman Spectroscopy* **43**, 571-576 (2012).

6. Casciani, F. & Condrate Sr, R. The Raman spectrum of monetite, CaHPO4. *Journal of Solid State Chemistry* **34**, 385-388 (1980).

7. Batool, S., Liaqat, U., Hussain, Z. & Sohail, M. Synthesis, characterization and process optimization of bone whitlockite. *Nanomaterials* **10**, 1856 (2020).

8. De Aza, P. et al. Vibrational properties of calcium phosphate compounds. 1. Raman spectrum of β-tricalcium phosphate. *Chemistry of materials* **9**, 912-915 (1997).

9. Rygula, A. et al. Raman spectroscopy of proteins: a review. *Journal of Raman Spectroscopy* **44**, 1061-1076 (2013).
